# The mitochondrial transporter SLC25A25 links ciliary TRPP2 signaling and cellular metabolism

**DOI:** 10.1371/journal.pbio.2005651

**Published:** 2018-08-06

**Authors:** Alexis Hofherr, Claudia Seger, Fiona Fitzpatrick, Tilman Busch, Elisabeth Michel, Jingting Luan, Lea Osterried, Frieder Linden, Albrecht Kramer-Zucker, Barbara Wakimoto, Conny Schütze, Nils Wiedemann, Anna Artati, Jerzy Adamski, Gerd Walz, Edmund R. S. Kunji, Craig Montell, Terry Watnick, Michael Köttgen

**Affiliations:** 1 Renal Division, Department of Medicine, Medical Center – University of Freiburg, Faculty of Medicine, University of Freiburg, Freiburg im Breisgau, Germany; 2 Spemann Graduate School of Biology and Medicine (SGBM), University of Freiburg, Freiburg im Breisgau, Germany; 3 Faculty of Biology, University of Freiburg, Freiburg im Breisgau, Germany; 4 Medical Research Council Mitochondrial Biology Unit, University of Cambridge, Cambridge, United Kingdom; 5 Department of Biology, University of Washington, Seattle, Washington, United States of America; 6 Institute of Biochemistry and Molecular Biology, ZBMZ, Faculty of Medicine, University of Freiburg, Freiburg im Breisgau, Germany; 7 BIOSS Centre for Biological Signalling Studies, University of Freiburg, Freiburg im Breisgau, Germany; 8 Institute of Experimental Genetics, Genome Analysis Center, Helmholtz Zentrum München, German Research Center for Environmental Health, Neuherberg, Germany; 9 Lehrstuhl für Experimentelle Genetik, Technische Universität München, Freising-Weihenstephan, Germany; 10 German Center for Diabetes Research (DZD), München-Neuherberg, Germany; 11 Neuroscience Research Institute and Department of Molecular, Cellular and Developmental Biology, University of California Santa Barbara, Santa Barbara, California, United States of America; 12 Division of Nephrology, University of Maryland School of Medicine, Baltimore, Maryland, United States of America; University of Pittsburgh, United States of America

## Abstract

Cilia are organelles specialized in movement and signal transduction. The ciliary transient receptor potential ion channel polycystin-2 (TRPP2) controls elementary cilia-mediated physiological functions ranging from male fertility and kidney development to left–right patterning. However, the molecular components translating TRPP2 channel–mediated Ca^2+^ signals into respective physiological functions are unknown. Here, we show that the Ca^2+^-regulated mitochondrial ATP-Mg/P_i_ solute carrier 25 A 25 (SLC25A25) acts downstream of TRPP2 in an evolutionarily conserved metabolic signaling pathway. We identify SLC25A25 as an essential component in this cilia-dependent pathway using a genome-wide forward genetic screen in *Drosophila melanogaster*, followed by a targeted analysis of SLC25A25 function in zebrafish left–right patterning. Our data suggest that TRPP2 ion channels regulate mitochondrial SLC25A25 transporters via Ca^2+^ establishing an evolutionarily conserved molecular link between ciliary signaling and mitochondrial metabolism.

## Author summary

Cilia are extensions of the cell that project from the cell body and serve as cellular sensors that transduce extracellular stimuli into the cell. The molecular mechanisms involved in ciliary signal transduction include ciliary receptors and ion channels. The transient receptor potential channel polycystin-2 (TRPP2) is known to control cilia-dependent biological processes throughout the animal kingdom ranging from male fertility to left–right organ patterning. However, the mechanisms mediating TRPP2 function in vivo remain unclear. Here, we have analyzed these mechanisms in the fruit fly and zebrafish and identified the mitochondrial solute carrier 25 A 25 (SLC25A25) as an intracellular effector of Ca^2+^-permeable TRPP2 cation channel in cilia. We show that SLC25A25 cooperates with TRPP2 in an evolutionarily conserved metabolic signaling pathway acting as a Ca^2+^-activated mitochondrial ATP transporter. Loss of this signaling pathway impairs cellular metabolism and causes severe defects in flies and fish, such as left–right patterning or male fertility problems. Our findings establish a molecular link between ciliary signaling and mitochondrial metabolism, raising the fascinating question of how SLC25A25-mediated metabolic fluctuations control male fertility and organ patterning in vivo.

## Introduction

Cilia are cellular appendages that coordinate several signaling pathways during development and tissue homeostasis [1]. Various signaling proteins, such as G protein–coupled receptors and transient receptor potential (TRP) channels, are compartmentalized to cilia to sense the cellular environment [2]. Yet little is known about how ciliary signals are propagated to control cellular functions.

TRP channels present in cilia have been proposed to regulate local Ca^2+^ levels [3,4]. Ca^2+^ signaling regulates many cellular processes, including mitochondrial function and cellular metabolism [5–7]. Hundreds of proteins have evolved to undergo conformational changes upon Ca^2+^ binding to control cellular signaling [7,8]. At the core of these versatile signaling pathways are Ca^2+^-permeable ion channels and Ca^2+^-regulated effector proteins. However, there is little known about the functional connection between specific channels and corresponding effector proteins.

**Fig 1:**
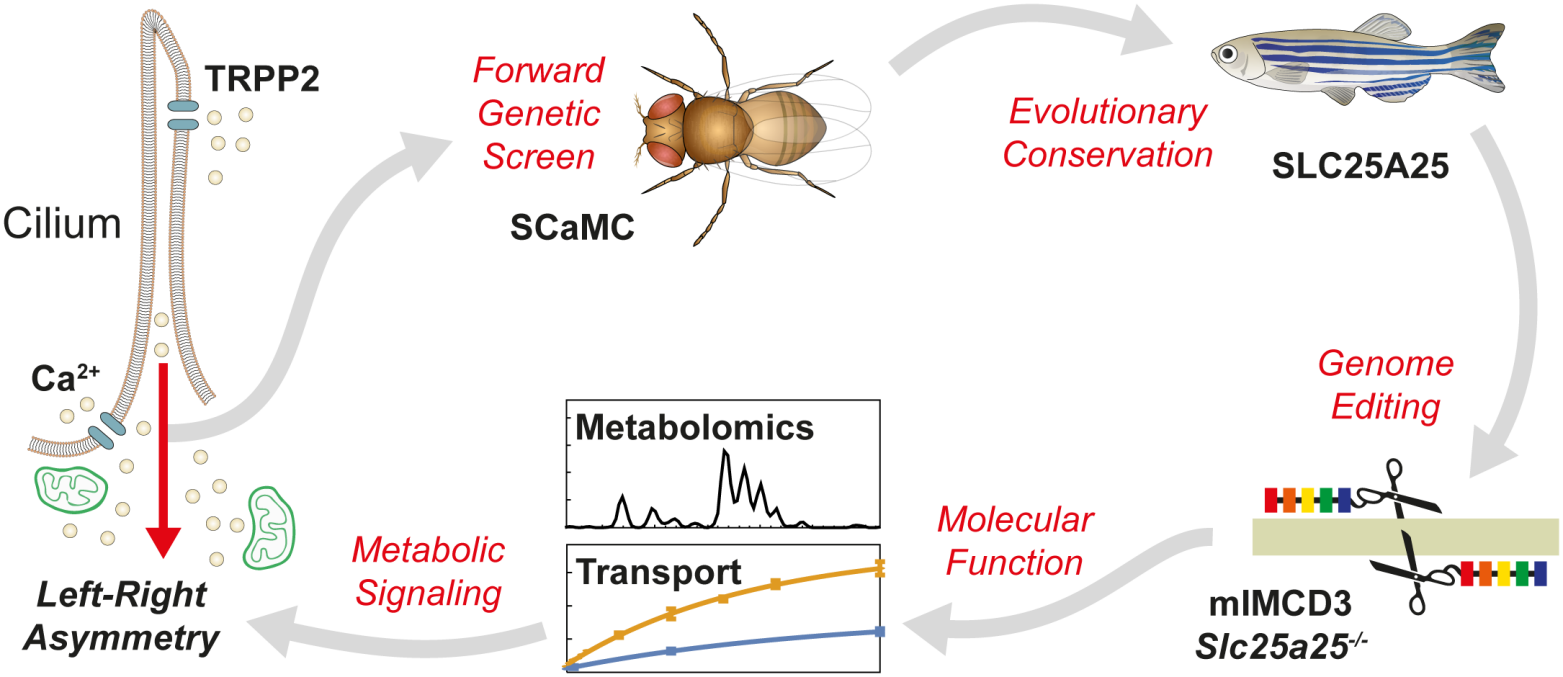
Schematic of the workflow to identify components of the TRPP2 signaling cascade. A forward genetic screen in *D*. *melanogaster* identified SCaMC as a potential downstream target of TRPP2. The evolutionary conservation of the SCaMC vertebrate homolog SLC25A25 in TRPP2-dependent signaling was validated in zebrafish. Genome editing, transport assays, and metabolomics were used to investigate the molecular function of SLC25A25 in vitro, suggesting a mechanistic link between cilia and mitochondria. SLC25A25, solute carrier 25 A 25; TRPP2, transient receptor potential channel polycystin-2.

Transient receptor potential channel polycystin-2 (TRPP2) is a ciliary Ca^2+^-permeable nonselective cation channel [9,10]. Loss of TRPP2 causes severe phenotypes throughout the animal kingdom, including polycystic kidney disease in humans, left–right patterning defects in vertebrate model organisms, and male infertility in invertebrates [9,11–14]. At the cellular level, TRPP2-mediated Ca^2+^ signals have been proposed to regulate processes such as transcription, proliferation, and differentiation [15–17]. However, the molecular components connecting TRPP2 to specific physiological functions have remained elusive. This raises the fundamental question of how ciliary TRPP2 ion channel activity controls processes like left–right patterning and fertility—or more specifically, what are the Ca^2+^-regulated proteins that act downstream of TRPP2 to control cellular metabolism? We have addressed this issue with a forward genetic screen in *D*. *melanogaster* followed by a targeted analysis of left–right pattering in *Danio rerio* (Fig 1).

## Results and discussion

### A genome-wide screen in *D*. *melanogaster* identifies the Ca^2+^-regulated mitochondrial carrier SCaMC in TRPP2 signaling

**Fig 2:**
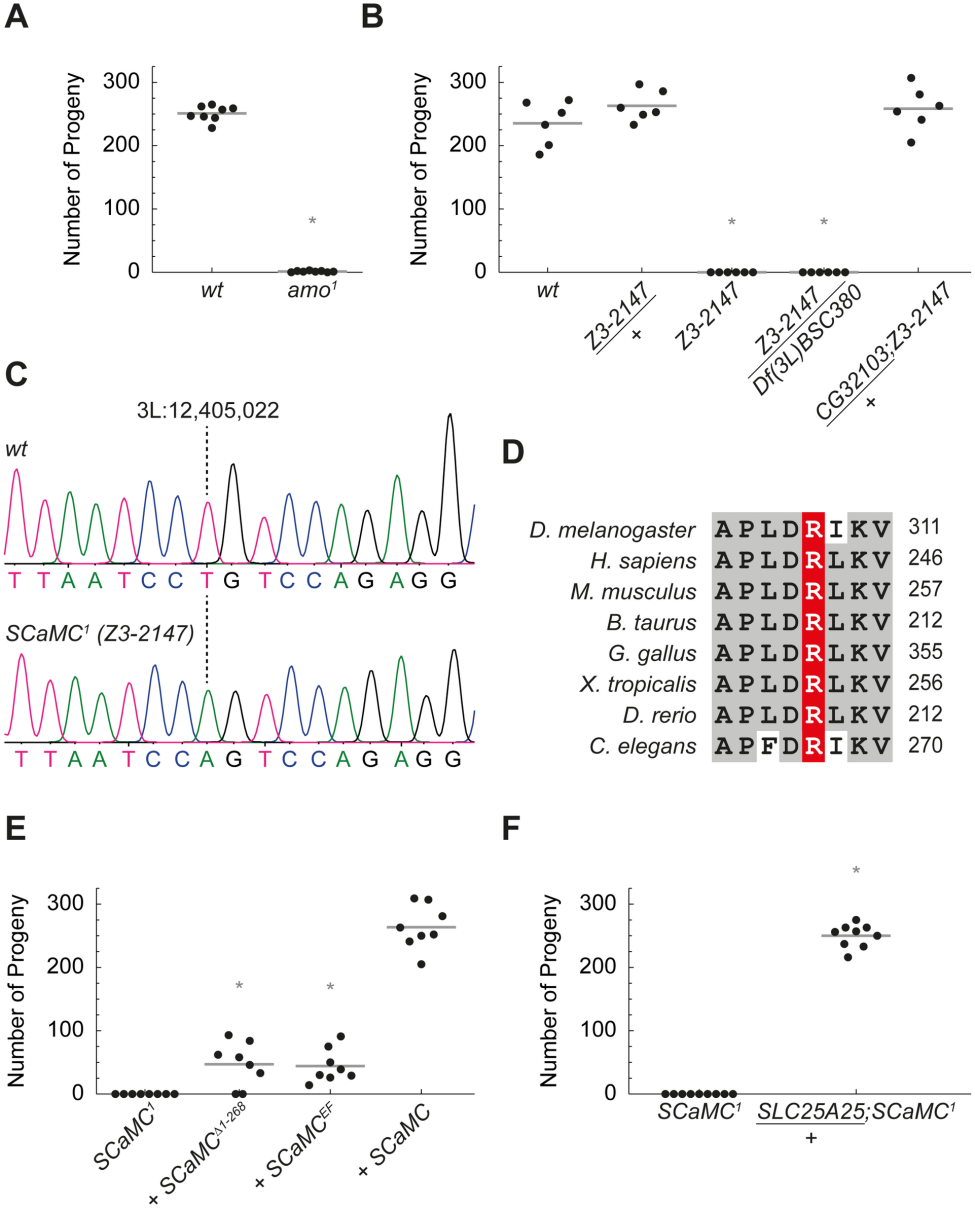
SCaMC is a Ca^2+^-regulated protein required for sperm storage in *Drosophila*. (A) *D*. *melanogaster* TRPP2 mutants (*amo*^*1*^) were male sterile (*P = 9 × 10^−11^). (B) Homozygous *Z3-2147* males and males with *Z3-2147* over a third-chromosomal deficiency deleting *CG32103*, *Df(3L)BSC380*, were infertile (both *P = 0.003). *Z3-2147/+* heterozygous males showed normal fertility. One copy of the *CG32103* cDNA fully rescued the fertility defect in *Z3-2147* homozygous males (*CG32103/+;Z3-2147*). (C) Sequencing chromatogram of *SCaMC*^*1*^ (*Z3-2147*) flies. Mutant flies presented with a point mutation on the third chromosome at position 3L:12,405,022, causing a change of thymine to adenine. (D) This missense mutation translates into replacement of basic arginine (R) 308 to nonpolar tryptophan (W) in the evolutionarily highly conserved first mitochondrial carrier domain of SCaMC (CG32103). (E) Mutations in the EF-hand domains of SCaMC (CG32103) impaired its function. *SCaMC* variants were tested in *SCaMC*^*1*^ (CG32103^R308W^) mutant background. While transgenic rescue with *SCaMC* wt cDNA (*+SCaMC*) behaved like wt males, transgenes lacking the N-terminal EF-hands (+*SCaMC*^*Δ1–268*^) or transgenes comprising missense mutations inactivating the Ca^2+^-binding EF-hand domains (+*SCaMC*^*EF*^) barely rescued *SCaMC*^*1*^ male fertility (both *P = 0.0009; compared to wt rescue). (F) Human SLC25A25 cDNA rescued infertility in *SCaMC*^*1*^ homozygous males (*SLC25A25/+; SCaMC*^*1*^) (*P = 0.0001). For numerical values, see S1 Data. SLC25A25, solute carrier 25 A 25; TRPP2, transient receptor potential channel polycystin-2; wt, wild-type.

We have previously shown that ciliary TRPP2 (Amo) is required for male fertility in flies [12,13]. Amo-deficient males produce motile sperm that are transferred to the uterus but do not reach the female sperm storage organs, which is critical for reproductive success (Fig 2A and S1 Movie). Based on this phenotype, we performed an unbiased forward genetic screen in flies to identify novel components of the TRPP2 signaling pathway in vivo (S1A Fig). Since we were interested in ciliary signaling rather than ciliary structure, we excluded mutations with defects in structure or motility of sperm. Out of a collection of 2,216 ethyl methanesulfonate–induced male sterile lines, we found that 404 produced mature sperm [18,19]. Among the latter set, 90 lines produced normal amounts of motile sperm but failed to be stored in the female sperm storage organs, similar to the *amo*^*1*^ mutant phenotype (S1B–S1E Fig) [12,13]. Using deficiency mapping and exon sequencing, we identified several of the corresponding genes. We focused our attention on the line *Z3-2147*, since the mutated gene *CG32103* contained Ca^2+^-binding EF-hands, suggesting a putative function downstream of TRPP2.

Similar to Amo-deficient males, homozygous *Z3-2147* males were infertile due to a sperm storage defect (Fig 2B and S1D–S1G Fig). We found a mutation in *CG32103* causing replacement of the conserved arginine 308 by a tryptophan in CG32103 (CG32103^R308W^) (Fig 2C and 2D). The deleterious impact of CG32103^R308W^ was validated by complementation experiments and full rescue of the male sterile phenotype with a transgene expressing wild-type *CG32103* cDNA (Fig 2B, S2A and S2B Fig). This demonstrates that the missense mutation CG32103^R308W^ causes the *Z3-2147* phenotype.

The function of *Drosophila* CG32103 is unknown [20]. The protein encoded by *CG32103* belongs to the family of evolutionarily conserved short Ca^2+^-binding mitochondrial carriers (SCaMC, or ATP-Mg/P_i_ carrier [APC]) (S3A–S3C Fig) [21,22]. The specific activation of these carriers by Ca^2+^ is based on conformational changes mediated by Ca^2+^ binding to the N-terminal EF-hands [23,24]. To test whether Ca^2+^ regulation of SCaMC (CG32103) is required in vivo, we performed rescue experiments comparing wild-type *SCaMC* and Ca^2+^ binding–deficient transgenes in *D*. *melanogaster*. In contrast to wild type, transgenes lacking the N-terminal EF-hands of *SCaMC* (*SCaMC*^*Δ1−268*^) or missense mutations abolishing Ca^2+^ binding (*SCaMC*^*EF*^) significantly impaired the rescue of the *SCaMC*^*1*^ (CG32103^R308W^) phenotype, suggesting that SCaMC activity is regulated through Ca^2+^ in vivo (Fig 2E and S2A–S2D Fig). The identical phenotypes of *amo*^*1*^ and *SCaMC*^*1*^ mutant flies in combination with the Ca^2+^ regulation of SCaMC support a model in which both proteins act in a common pathway.

### Solute carrier 25 A 25 (SLC25A25), the vertebrate ortholog of *D*. *melanogaster* SCaMC, is a Ca^2+^-regulated ATP transporter

To investigate the evolutionary conservation of SCaMC function, we tested whether its human homolog SLC25A25 could substitute for *Drosophila* SCaMC. Human *SLC25A25* fully rescued the male infertility in *SCaMC*^*1*^ flies (Fig 2F).

**Fig 3:**
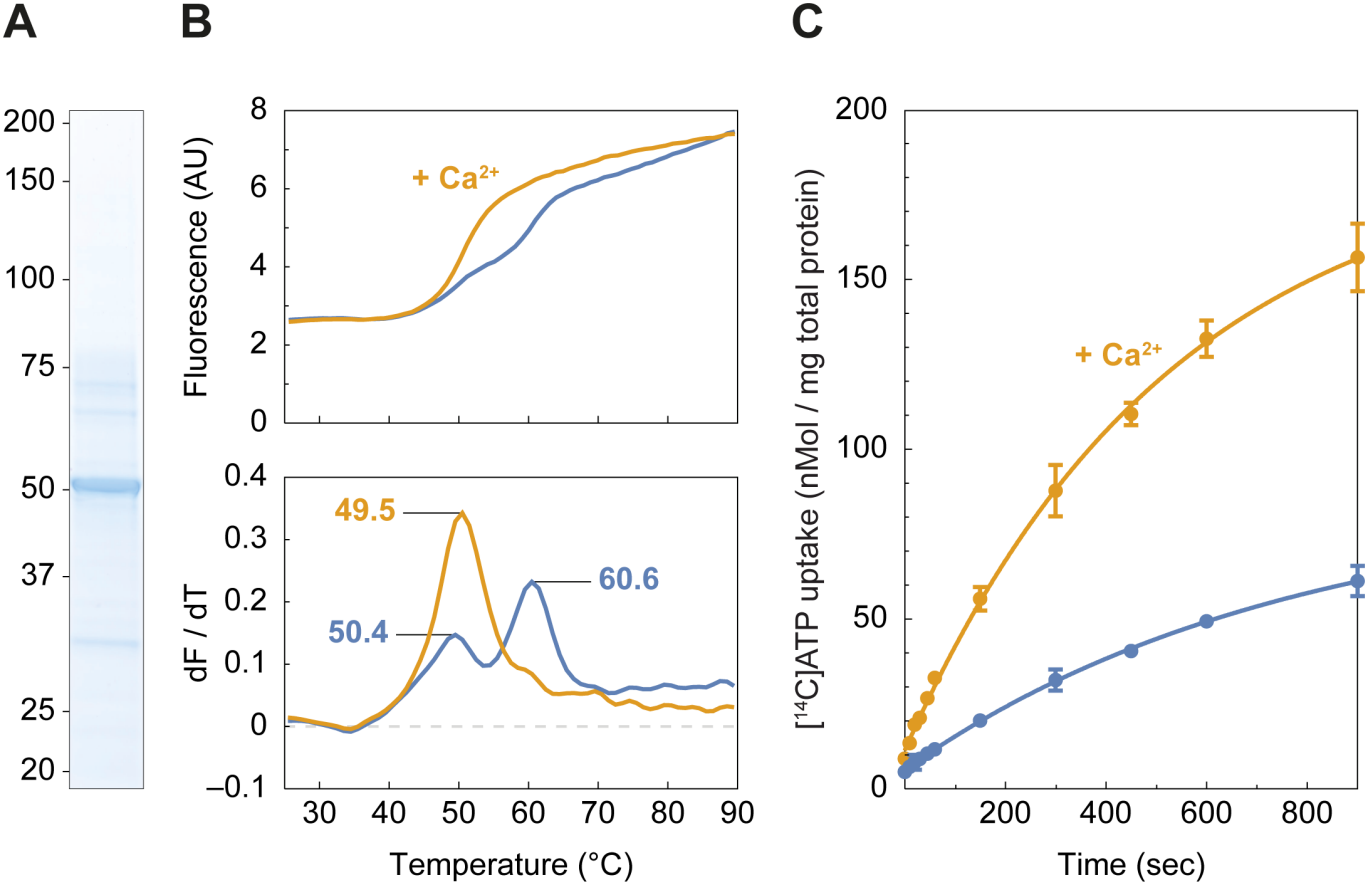
SLC25A25 is a Ca^2+^-regulated Mg-ATP transporter. (A) Purified SLC25A25 (5 μg) was separated by SDS-PAGE and visualized by Coomassie staining. (B) Thermostability profile (top) and its corresponding first derivative (bottom) of SLC25A25. Assays were performed in triplicate with the addition CaCl_2_ (orange line) or without (blue line). (C) Uptake assays of [^14^C]-labeled Mg-ATP into proteoliposomes with the addition CaCl_2_ (orange line) and without (blue line). The error bars represent the standard deviation of 4 replicates, and the uptake curves were fitted with a one-phase association curve (r-squared > 0.99). For numerical values, see S1 Data. AU, arbitrary unit; SLC25A25, solute carrier 25 A 25.

*Drosophila* SCaMC and SLC25A25 share similar transport and Ca^2+^-binding domains, but like SCaMC, the molecular function of human SLC25A25 has not been established. SLC25A25 is closely related to the Ca^2+^-regulated exchanger of cytosolic adenine nucleotides for phosphate (APC) from the mitochondrial matrix [21,22]. To test this hypothesis and determine the function and regulation of SLC25A25, we purified the transporter protein and characterized it in vitro (Fig 3A). Thermostability assays were used to assess the effect of Ca^2+^ on the apparent melting temperature (*T*_*m*_) of SLC25A25 (Fig 3B). A biphasic unfolding profile was observed, indicating that SLC25A25 was in a mixed population consisting of a Ca^2+^-bound state (apparent *T*_*m*_ = 49.5 °C) and a Ca^2+^-free state (apparent *T*_*m*_ = 60.6 °C), with similar values to those observed for the paralog SLC25A24 [24]. After addition of Ca^2+^, the apparent *T*_*m*_ shifted to a single peak (apparent *T*_*m*_ = 50.4 °C), indicating that the mixed population had shifted to a homogeneous population of Ca^2+^-bound protein. These results indicate that SLC25A25 undergoes a Ca^2+^-dependent conformational change (Fig 3B). To test whether Ca^2+^ regulates the transport activity and whether SLC25A25 transports Mg-ATP, purified transporter protein was reconstituted into proteoliposomes, and the uptake of radio-labeled [^14^C]-Mg-ATP was monitored with and without added Ca^2+^. Upon addition of Ca^2+^, a significant increase in the uptake rate of [^14^C]-Mg-ATP was observed, showing that the SLC25A25 transport activity was up-regulated by Ca^2+^ (Fig 3C). Taken together, these experiments demonstrate that SLC25A25 is a Ca^2+^-regulated Mg-ATP transporter.

### SLC25A25 is required for left–right organ patterning in zebrafish

**Fig 4:**
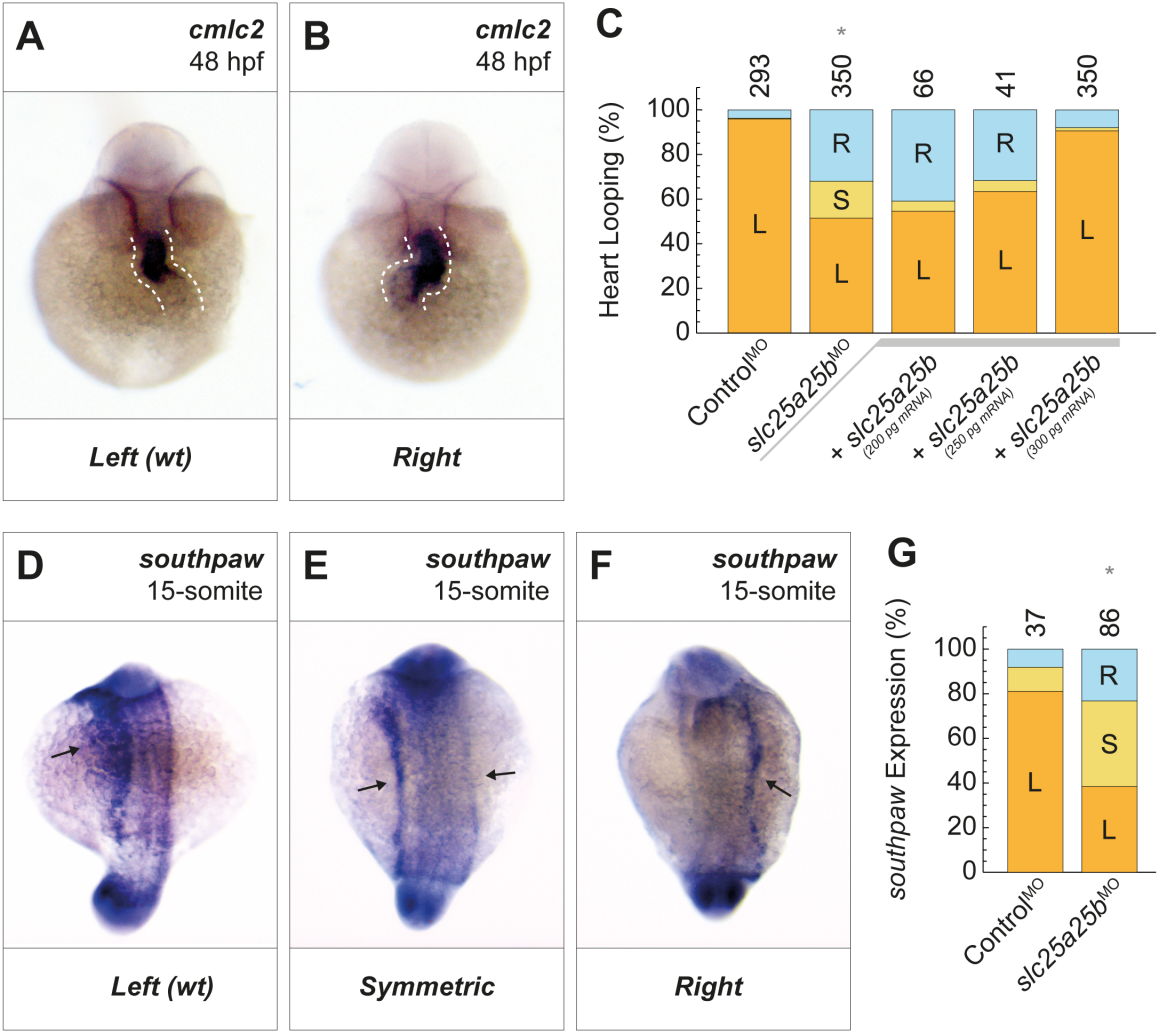
*slc25a25b* is required for left–right organ patterning in zebrafish. (A,B) As a measure for left–right asymmetry, heart looping of *slc25a25b*-morphant fish was visualized by in situ hybridization for *cmlc2*. (C) Knockdown of *slc25a25b* caused randomization of heart looping (*slc25a25b*^*MO*^; *P = 1.1 × 10^−34^). This phenotype was rescued by injection (+) of *slc25a25b* mRNA in a concentration-dependent fashion (*+slc25a25b*). (D-F) *southpaw* (*nodal*) expression in 15-somite stage *slc25a25b*-morphant zebrafish embryos was visualized by in situ hybridization. (G) In contrast to control fish, in which *southpaw* expression was largely restricted to the left side, *slc25a25b*-morphant fish showed randomized *southpaw* expression (*P = 0.00008). Numbers of embryos are indicated above bars. L = left; S = symmetric; R = right. This denomination for heart looping is equivalent to wt *d-loop*, symmetric *no-loop*, and reversed, sinistral *s-loop* [33]. For numerical values, see S1 Data. *cmlc2*, *cardiac myosin light chain 2*; hpf, hours post fertilization; wt, wild type.

We addressed the question whether SLC25A25 function is required in vertebrate TRPP2 signaling, using zebrafish as a model organism. In zebrafish, Trpp2 is required for left–right patterning (S4A–S4D Fig) [25–29]. Rotating motile cilia in the left–right organizer generate a fluid flow-mediated directional signal that is sensed by primary cilia [30,31]. Ciliary TRPP2 is required to translate this signal into correct left–right patterning [9,11,32]. Here, we show that loss of Slc25a25b (*slc25a25b*) phenocopied loss of Trpp2 (*pkd2*), resulting in randomization of left–right asymmetry (Fig 4A–4C and S5A–S5F Fig). Similar to loss of Trpp2, number of cilia, ciliary length, and cilia-dependent directional flow generation in the zebrafish left–right organizer (Kupffer’s vesicle [KV]) were not affected by loss of Slc25a25b (S6A, S6B and S7A–S7H Figs).

### SLC25A25 regulates left–right patterning upstream of Nodal

Asymmetric expression of members of the Nodal cascade is required for left–right patterning [34,35]. TRPP2-mediated Ca^2+^ signaling has been reported to control this cascade through an unknown mechanism [11,27,32,36]. We, therefore, tested whether SLC25A25 represents a missing link connecting asymmetric TRPP2 activity to Nodal. In support of this hypothesis, knockdown of *slc25a25b* resulted in randomization of *southpaw* (*Nodal*) expression in the lateral plate mesoderm (Fig 4D–4G and S8A–S8D Fig). Furthermore, other members of the Nodal signaling cascade were affected by *slc25a25b* knockdown, including *dand5* (*Cerl2*) and *lefty2* (S8E–S8K Fig). These observations demonstrate that *slc25a25b* acts upstream of the Nodal cascade, providing a molecular link between TRPP2-mediated Ca^2+^ signals and asymmetric gene expression.

### TRPP2 and SLC25A25 are compartmentalized in microdomains

**Fig 5:**
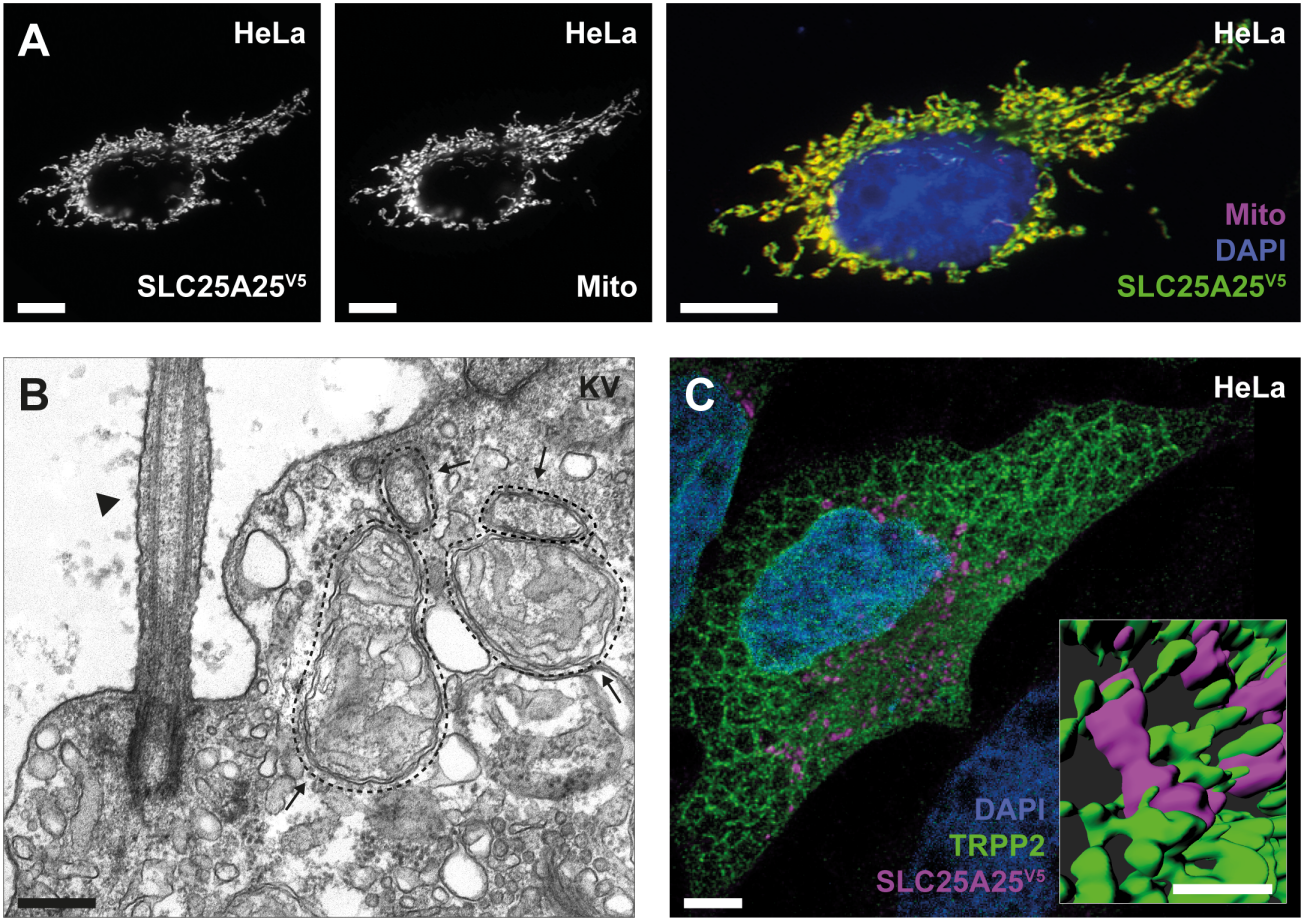
TRPP2 and SLC25A25 form a signaling microdomain. (A) Indirect immunofluorescence of SLC25A25^V5^ showed colocalization with the mitochondrial marker pDsRed2-Mito (scale bar = 10 μm). (B) Transmission electron microscopy of a ciliated cell from KV (arrowhead: cilium; arrows: mitochondria; scale bar = 200 nm). Representative of 3 images with a similar pattern. (C) Indirect immunofluorescence of SLC25A25^V5^ (magenta) and TRPP2 (green) showed tight spatial coupling of both proteins at ER–mitochondria junctions in epithelial cells using STED microscopy (scale bar = 5 μm; inset = 1 μm). The inset shows 3D rendering of a magnification depicting close contacts between the ER and mitochondria (scale bar = 1 μm). ER, endoplasmic reticulum; KV, Kupffer’s vesicle; SLC25A25, solute carrier 25 A 25; SLC25A25^V5^, V5-tagged SLC25A25; STED, stimulated emission depletion; TRPP2, transient receptor potential channel polycystin-2.

Mechanistically, our data support a model in which TRPP2-mediated Ca^2+^ signals regulate SLC25A25 activity via its four EF-hand domains (Figs 2E and 3, S3A Fig). Ca^2+^ is a promiscuous second messenger regulating many different cellular processes. Specificity of Ca^2+^ signals is achieved through tight spatial coupling of the local Ca^2+^ source and Ca^2+^-dependent effector proteins in microdomains [8]. Ca^2+^-permeable TRPP2 channels are found in primary cilia and in the endoplasmic reticulum (ER), whereas SLC25A25 is expressed in the inner mitochondrial membrane (Fig 5A) [37,38]. Close proximity of these cellular compartments is required for functional coupling of TRPP2 and SLC25A25 in a signaling microdomain. We found that mitochondria cluster at the ciliary base in KV (Fig 5B and S9A Fig) [39]. Hence, we suggest that TRPP2-mediated Ca^2+^ influx at the ciliary base may control SLC25A25 activity. Furthermore, we observed that ER-resident TRPP2 and mitochondrial SLC25A25 showed tight spatial coupling in epithelial cells (Fig 5C and S9B Fig). This is in line with the close contacts between the ER and mitochondria at mitochondria-associated ER membranes [40]. In the ER, which is the main intracellular Ca^2+^ store, TRPP2 contributes to Ca^2+^ release and may amplify ciliary Ca^2+^ signals [3,41]. Ca^2+^ release from the ER results in mitochondrial Ca^2+^ transients that regulate cellular metabolism [40]. Taken together, the proximity of TRPP2 and SLC25A25 supports the functional interaction of both proteins. In *D*. *melanogaster* sperm, TRPP2 is exclusively expressed in the ciliary membrane and SCaMC in mitochondria. Therefore, the critical cellular location for the TRPP2/SCaMC interaction is the cilia/mitochondria interphase (S1F–S1G, S3B and S3C Figs). The additional ER expression of TRPP2 in vertebrate cells, on the other hand, precludes an unambiguous localization of the functional coupling of SLC25A25 and TRPP2 to an ER/mitochondria or cilia/mitochondria microdomain.

### Modulation of mitochondrial metabolism by SLC25A25 and TRPP2

**Fig 6:**
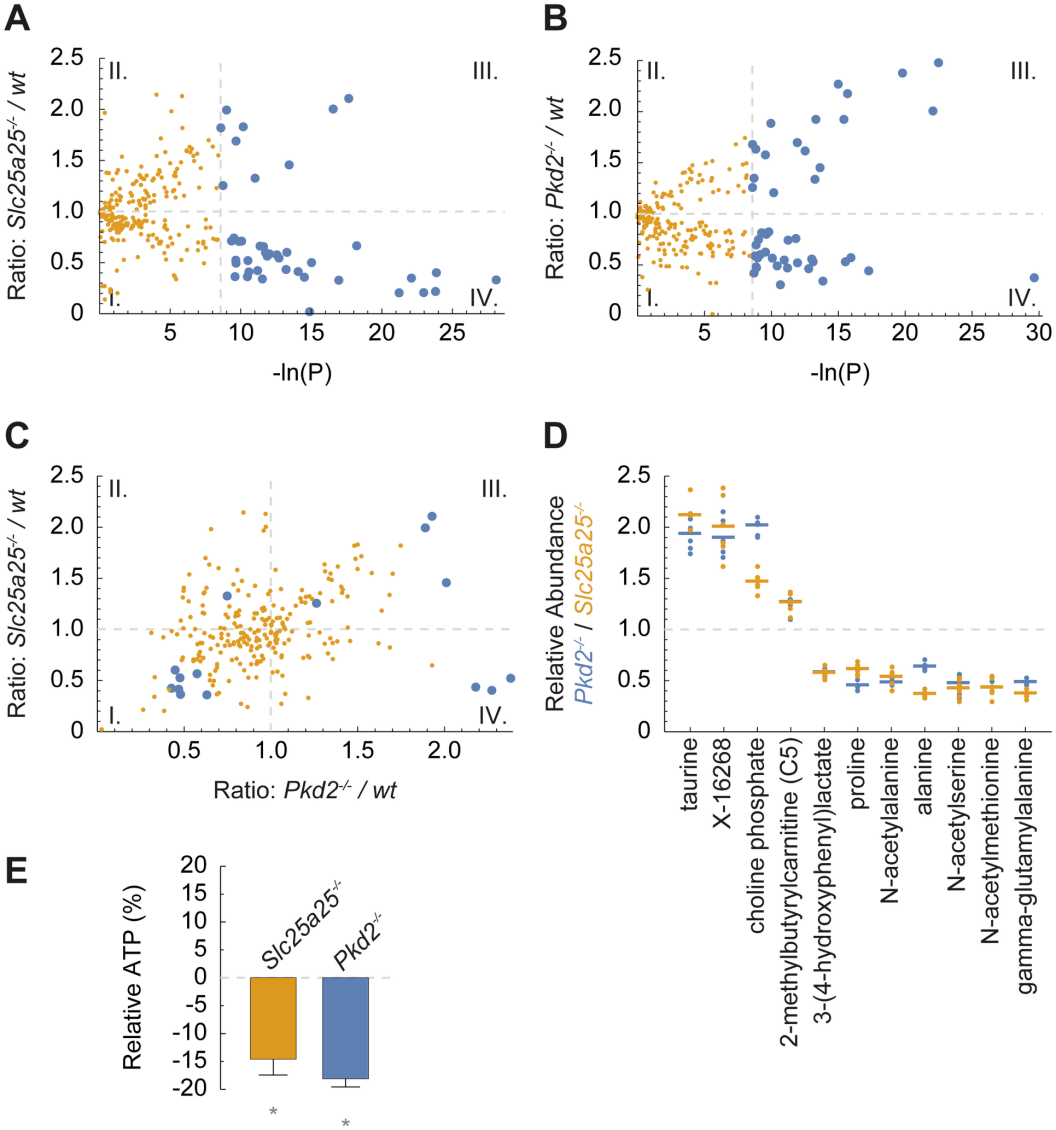
Metabolic profiles of TRPP2- and SLC25A25-deficient cells. (A) The ratio of metabolites in *Slc25a25*-deficient and wt mIMCD3 cells are plotted against their *P* value (each dot represents 1 metabolite). The analysis showed 42 significantly changed metabolites in *Slc25a25*-deficient cells (blue dots; *n* = 6, *P ≤ 0.00019). (I.) decreased, not significant; (II.) increased, not significant; (III.) significantly increased; and (IV.) significantly decreased. (B) Statistical evaluation of metabolite ratios comparing mIMCD3 *Pkd2*^*−/−*^ and wt cells. The analysis showed 55 significantly changed metabolites in *Pkd2*-deficient cells (blue dots; *n* = 6, *P ≤ 0.00019). (C) Ratios of metabolites of SLC25A25-deficient and TRPP2-deficient cells (*Pkd2*^*−/−*^) relative to wt cells showed concordant changes of several metabolites. Four metabolites were significantly increased (III.) and 7 significantly decreased (I.) in both knockout cell lines (blue dots; *n* = 6, *P ≤ 0.00019). (D) Relative abundance of metabolites with concordant changes in TRPP2- and SLC25A25-deficient cells compared to wt cells (metabolites from quadrant I. and III. in panel A). (E) Cellular ATP content was decreased in SLC25A25- and TRPP2-deficient cells (depicted as relative change compared to wt cells; *n* = 6, *P ≤ 0.01). For numerical values, see S2 Data. SLC25A25, solute carrier 25 A 25; TRPP2, transient receptor potential channel polycystin-2; wt, wild type.

The evolutionary conservation of SLC25A25 in TRPP2-dependent signaling raises the question how a mitochondrial carrier controls distinct cellular outcomes. An important prerequisite to understanding the physiological role of a mitochondrial carrier protein is the clarification of its impact on cellular metabolism [42]. To elucidate the role of SLC25A25-mediated transport in cellular metabolism, we performed metabolomic analyses and measured mitochondrial oxidative metabolism. We generated SLC25A25- and TRPP2-deficient epithelial cells using genome editing (*Slc25a25*^*−/−*^ and *Pkd2*^*−/−*^) (S10A–S10E Fig) [43]. We analyzed Ca^2+^ signaling and mitochondrial oxidative metabolism in wild-type and knockout cells (S11A and S11B Fig). SLC25A25-deficient cells showed a decrease in overall cellular respiration that did not affect growth rate or survival (S11C Fig) [44]. To identify SLC25A25-dependent metabolites, we performed broad-coverage discovery metabolomics encompassing the entire metabolome (S2 Data) [45]. In SLC25A25-deficient cells, 42 metabolites were significantly altered compared to wild-type cells (Fig 6A).

The model that TRPP2 and SLC25A25 operate in a common pathway predicts that loss of TRPP2 channel activity or loss of SLC25A25 carrier function results in concordant changes in specific metabolites. We therefore analyzed the metabolite profiles of TRPP2-deficient cells (Fig 6B and S2 Data). We found 4 metabolites that were significantly increased and 7 metabolites that were significantly decreased in both TRPP2- and SLC25A25-deficient cells (Fig 6C and 6D and S1 Table). Furthermore, ATP concentrations were reduced in SLC25A25- and TRPP2-deficient cells (Fig 6E). This observation is in line with the reported role of the SCaMC/APC carrier family in modulating the adenine nucleotide pool in the mitochondrial matrix in response to changes in energy demands [22,46,47].

Emerging evidence suggests that metabolite fluctuations regulate cellular signal transduction [48]. Several solute carriers have been implicated in metabolic signaling [42,49]. Consequently, we propose that activation of the TRPP2-SLC25A25 pathway induces downstream metabolic signals. In our experiments, we found that significantly changed metabolites are associated with amino acid metabolism, including branched-chain amino acids (BCAAs) in mitochondria (S1 Table). Several of the metabolites we discovered are involved in cellular signal transduction, but their role in *Drosophila* sperm movement and zebrafish left–right patterning has not been explored yet. Notably, ATP has been implicated in both processes. Sperm motility is an ATP-dependent process. Directional sperm movement in *Drosophila* depends on TRPP2 [12,13]. Our data suggest that TRPP2-mediated Ca^2+^ signals activate SLC25A25-dependent adenine nucleotide import, which leads to an increase in ATP output from the mitochondrion to increase cytosolic ATP, which drives sperm motility via dynein motor proteins. In left–right patterning, it has been proposed that ATP is released from the left–right organizer to promote the spreading of left-sided Nodal signaling to the lateral plate mesoderm via purinergic signaling [50]. A similar purinergic signaling mechanism has been shown for TRPP2-mediated ciliary signaling in epithelial cells [51]. Ultimately, TRPP2 activity in the left–right organizer causes asymmetric gene expression of the Nodal cascade [11,32]. This transcriptional regulation might be explained by the observed metabolic changes, since almost all chromatin-modifying enzymes utilize metabolites as cofactors to control gene expression [52]. However, the precise molecular mechanism linking SLC25A25-mediated metabolic fluctuations and asymmetric gene expression in the left–right organizer remains to be determined.

### SLC25A25 is an evolutionarily conserved downstream effector of TRPP2 signaling linking ciliary signaling and cellular metabolism

**Fig 7:**
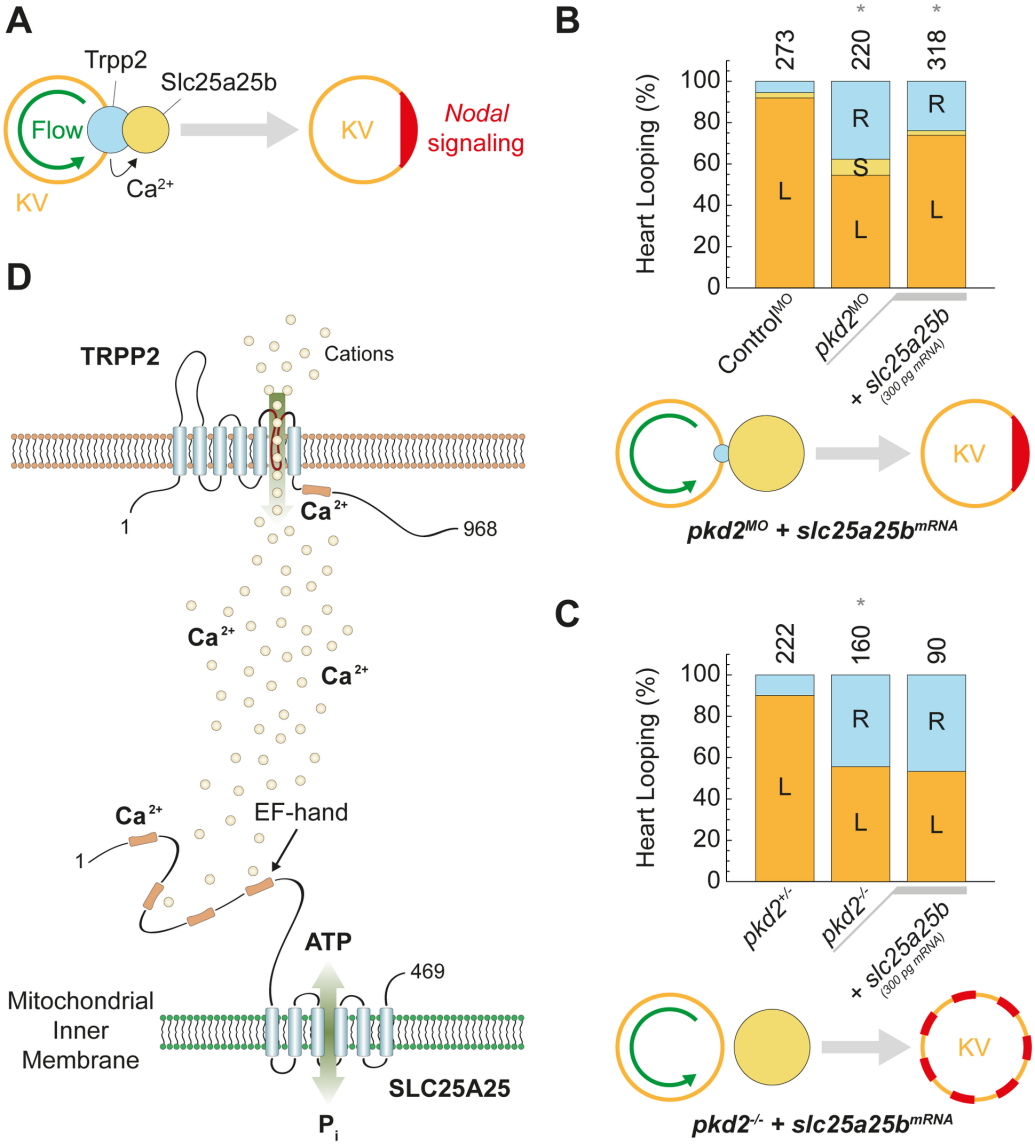
TRPP2 and SLC25A25 act in a common signaling cascade required for vertebrate left–right patterning. (A) Conceptually, our data imply a function of SLC25A25 (Slc25a25b) between flow-mediated asymmetric activation (green) of TRPP2-dependent Ca^2+^ signaling in KV and Nodal-dependent asymmetric gene expression in the lateral plate mesoderm (red). (B) Randomized left–right asymmetry in *pkd2*-morphant zebrafish (*pkd2*^*MO*^; *P = 6 × 10^−21^) was partially rescued by expression of *slc25a25b* mRNA (*P = 4 × 10^−6^). (C) Randomized left–right asymmetry in *pkd2-*null (*pkd2*^*−/−*^) zebrafish (*P = 9 × 10^−15^) was not rescued by expression of *slc25a25b* mRNA (*P = 0.27). Numbers of embryos are indicated above bars. L = left; S = symmetric; R = right. (D) Schematic of functional coupling of TRPP2 and SLC25A25. For numerical values, see S1 Data. KV, Kupffer’s vesicle; SLC25A25, solute carrier 25 A 25; TRPP2, transient receptor potential channel polycystin-2.

Finally, to test whether SLC25A25 acts downstream of TRPP2 in vertebrate left–right patterning, we evaluated TRPP2-dependent SLC25A25 function in zebrafish (Fig 7A). Parallel knockdown of *slc25a25b* and *pkd2* did not aggravate the left–right patterning defect compared to individual suppression of either transcript, further supporting a role of both proteins in a common pathway (S12 Fig). Consequently, we performed rescue experiments overexpressing Slc25a25b in the Trpp2*-*deficient background. Slc25a25b expression significantly alleviated the loss of TRPP2 phenotype, suggesting a role of SLC25A25 downstream of TRPP2 (Fig 7B). TRPP2 is thought to be activated asymmetrically in the vertebrate left–right organizer, raising the question how symmetric expression of *slc25a25b* can rescue laterality in *pkd2*-morphant zebrafish (*pkd2*^*MO*^) [27,32]. In *pkd2* morphants, expression of Slc25a25b may rescue laterality by amplifying residual asymmetric Trpp2 activity (Fig 7B). If SLC25A25 and TRPP2 act in a common pathway, Slc25a25b should not rescue Trpp2-dependent left–right determination in a *pkd2*-null background (*pkd2*^*−/−*^) [25]. Indeed, expression of *slc25a25b* in *pkd2*-null zebrafish did not rescue left–right patterning, corroborating a role for SLC25A25 downstream of TRPP2 (Fig 7C).

In summary, our results establish an evolutionarily conserved link between the ciliary ion channel TRPP2 and the mitochondrial transporter SLC25A25. The identification and molecular characterization of the Ca^2+^-regulated mitochondrial ATP carrier SLC25A25 downstream of TRPP2 provides insights into the molecular mechanisms of signal transduction in cilia-dependent biological processes ranging from male fertility to vertebrate morphogenesis.

## Materials and methods

### Ethics statement

All animal experiments were conducted according to international guidelines and the German law for the welfare of animals. Approval for animal studies was obtained from the regional authorities (Regierungspräsidium Freiburg, reference number G-16/89) [53].

### Molecular biology

Full-length human *PKD2* (GenBankTM accession no. U50928) in pcDNA3 (Invitrogen) has been described previously [54]. *CG32103* cDNA was obtained from the Drosophila Genomics Resource Center, *Homo sapiens SLC25A25* (NM_052901) and *D*. *rerio slc25a25b* cDNA from BioCat. *V5-SCaMC*, *V5-SCaMC*^*Δ1–268*^, *V5-SCaMC*^*EF*^ (EF = D125/159/190/226A), [55] and *SLC25A25-V5* were generated by PCR-based mutagenesis. cDNA constructs were cloned into pGMR-β2tubulin for fly transgenesis [56]. The β2 tubulin promoter of pGMR-β2tubulin was used to drive transgene expression, pcDNA6 (Invitrogen) for cellular transfection, and a modified pXT7 (VF10) plasmid for in vitro transcription [57]. All DNA constructs were validated by Sanger sequencing.

### Antibodies

Rabbit anti-acetyl-α-tubulin (Lys40) (D20G3) (#5335; Cell Signaling), chicken anti-GFP (ab13970; Abcam), mouse anti-Pyruvate dehydrogenase E2/E3bp (ab110333; Abcam), goat anti-TRPP2 (G-20; Santa Cruz Biotechnology), rabbit anti-V5 epitope tag (Merck Millipore), and mouse anti-V5-Tag (Clone SV5-Pk1; Bio-Rad) antibodies were obtained commercially. Monoclonal anti-TRPP2^698–799^ antibodies and the anti-Amo antiserum have been described previously [12,58]. Western blot detection was performed using an anti-mouse (Dako) or anti-rabbit (GE Healthcare) horseradish peroxidase–coupled secondary antibody. Antigens were visualized by immunofluorescence using secondary goat anti-chicken Alexa 488 (Invitrogen), donkey anti-mouse Alexa 488 (Invitrogen), donkey anti-rabbit Alexa 488 (Invitrogen), and donkey anti-mouse Cy3 (Jackson Immunoresearch) antibodies.

### Cell culture and transfection

HeLa and mIMCD3 cells were obtained from the American Type Culture Collection. Cells were cultivated as adherent monolayers in DMEM (Lonza) and DMEM F-12 (Lonza) media supplemented with 10% heat-inactivated fetal bovine serum (FBS; Biochrom). Cell lines were maintained in a humidified 10% CO_2_ incubator at 36.5 °C. Cells were passaged every 3–4 d, using 0.05% and 0.25% Trypsin-EDTA (Gibco), respectively. Cells were transfected using Lipofectamine 2000 (Invitrogen) for HeLa and Cell Line Nucleofector Kit R (Lonza) for mIMCD3 cells.

### Construction of *Saccharomyces cerevisiae* expression strains and site-directed mutagenesis

The *slc25a25 isoform b* gene was codon-optimized for expression in *S*. *cerevisiae* (Genscript). The gene was truncated Δ1–15, and an 8-histidine tag followed by a Factor Xa cleavage site (IEGR) was introduced to the N-terminus of the gene. Vectors were isolated by miniprep (Real Genomics), and the gene was confirmed by sequencing (Source Bioscience). The gene was cloned into a modified *S*. *cerevisiae* expression vector pYES3/ CT, with the inducible galactose promoter replaced by a constitutively active promoter for the *S*. *cerevisiae* phosphate carrier *PIC2*, as described previously [59]. Expression vectors were transformed into the *S*. *cerevisiae* W303-1B strain based on the LiAc/SS carrier DNA/PEG method [60]. Transformed cells were selected on synthetic complete tryptophan dropout (SC-Trp) plates supplemented with 2% glucose.

### Growth of *S*. *cerevisiae* and mitochondrial isolation

A 5-liter preculture of SC-Trp medium (Formedium) supplemented with 2% glucose media was used to inoculate 50 L of YPD in an Applikon bioreactor. For large-scale cultures, cells were harvested by continuous centrifugation (CEPA Z41). Mitochondria were prepared using established methods, snap frozen in liquid nitrogen, and stored at −80 °C [61].

### Solubilization and protein purification of SLC25A25

The mitochondria were solubilized and purified by nickel affinity chromatography as described previously [23], with the following amendments: The nickel sepharose resin (GE Healthcare) was washed with 50 column volumes of buffer A containing 50 mM HEPES, pH 7.4, 200 mM sodium chloride, 50 mM imidazole, 0.1% (w/v) lauryl maltose neopentyl glycol, 0.1 mg/mL tetraoleoyl cardiolipin, and 30 column volumes of buffer B (Buffer A without imidazole). Cleavage of the protein from the histidine tag was carried out using Factor Xa protease (New England Biolabs) for 2.5 h at 10 °C. The purified protein was incubated with 10 mM EGTA overnight at 4 °C, and EGTA was removed using a PD10 desalting column (GE Healthcare).

### SDS-PAGE analysis of SLC25A25

Proteins were separated by using 4%–12% TruPAGE precast gels SDS-PAGE gels (Merck KGaA) loaded with 5 μg of protein at a 3:1 mix of sample to loading buffer and stained using Instant*Blue* Coomassie according to manufacturer’s instructions (Merck KGaA). Protein concentration was determined using a Spectrophotometer (NanoDrop ND-1000, NanoDropTechnologies) at absorbance 280 nm.

### Thermostability assays

A microscale method, based on the thiol reactive fluorophore N-[4-(7-diethylamino-4-methyl-3-coumarinyl)-phenyl]-maleimide (CPM), was used to measure the thermostability of SLC25A25 [62,63]. CPM dissolved at 5 mg/ml in DMSO was diluted into assay buffer (purification buffer B) and incubated at room temperature in the dark for 10 min. The protein stock was diluted to 40 μg/ml in the assay buffer (±1 mM CaCl_2_) and incubated on ice for 10 min. CPM working solution (5 μL) was added, and the sample was vortexed briefly and incubated at 4 °C in the dark for a further 10 min. Data were collected using a high-resolution melt (HRM) channel on a RotorGene Q 2plex HRM qPCR cycler with a 36-sample rotor (Qiagen). Measurements were made every 15 s at 1 °C intervals from 25–90 °C. Protein denaturation profiles were analyzed using the Rotor-gene Q software, and the peak in the derivative of the fluorescence was plotted from which the apparent melting temperature (*T*_*m*_), a relative measure of protein stability, was determined.

### Liposome preparation, reconstitution, and ATP transport assays

L-α-phosphatidylcholine (Avanti Polar Lipids) and tetraoleoyl cardiolipin (Avanti Polar Lipids) were mixed in a 20:1 (w/w) ratio and dried under a stream of nitrogen. The lipid mixture was washed with 500 μL of methanol and dried under nitrogen.

Approximately 65 μg of SLC25A25 in LMNG was reconstituted into liposomes loaded with 20 mM HEPES, pH 7.4, 1 mM DTT, 1 mM MgCl_2_, and 1 mM ATP as the internal buffer. The detergent pentaethylene glycol monodecyl ether was added to a final concentration of 1.6% (v/v), and the lipids were solubilized by vortexing and incubated on ice for 30 min. The protein was added, and the samples were incubated on ice for 5 min. The pentaethylene glycol monodecyl was removed by multiple additions of SM-2 bio-beads (Bio-Rad). Four additions of 60 mg and 1 addition of 480 mg of bio-beads were added to the sample every 20 min with inversion at 4 °C. The samples were incubated overnight at 4 °C with inversion. Bio-beads were removed by passage of the sample through empty micro-bio spin columns (Bio-Rad). The external substrate was removed using a PD10 desalting column (GE Healthcare).

Transport assays were carried out with a Hamilton MicroLab Star robot (Hamilton Robotics). One hundred μL of proteoliposomes in external buffer was loaded per well in a MultiScreen_HTS_-HA 96-well filter plate (pore size = 0.45 μm; Millipore). Uptake assays of radiolabeled ATP in the presence or absence of 1 mM CaCl_2_ were initiated by the addition of 100 μl of HEPES buffer with 2 μM [^14^C]-ATP (Perkin Elmer) per well. The uptake of [^14^C]-ATP was stopped after 0, 10, 20, 30, 45, 60, and 150 s and 5, 7.5, and 10 min incubation times with 200 μl ice-cold HEPES buffer, and the samples were filtered with a vacuum manifold, followed by 2 additional wash steps with 200 μl ice-cold HEPES buffer. Plates were dried overnight. Radioactivity was measured by adding 200 μl MicroScint-20 (Perkin Elmer) and quantification using a TopCount scintillation counter (Perkin Elmer). Uptake curves were fitted according to the one-phase association model (GraphPad, Prism).

### Transcription activator-like effector nucleases (TALENs)

TALEN target sites in mouse *Pkd2* and *Slc25a25* were chosen to generate loss-of-function deletions. Two TALEN pairs per gene were designed to induce parallel 5′ and 3′ genomic DNA double-strand breaks facilitating the deletion of the respective gene by nonhomologous end-joining [64]. TALEN binding sequences are

Pkd2-1: TCAGTAAATGCAGAGAGGA [43];
Pkd2-2: TACATGTAGAGTCAGTCCA [43];
Pkd2-3: TCTCTCATGCTTGATCACC [43];
Pkd2-4: TTCTGCTTTTCATAGGTTA [43];
Slc25a25-1: TGGTTAGAGCTGGTATCCC;
Slc25a25-2: TGACGGTACTGCCATTCTG;
Slc25a25-3: TTCCATCTCGTTCACTCAG;
Slc25a25-4: TGGACCAGAGGGATGCCGT.

TALENs were assembled using a previously described Golden Gate assembly kit [65,66]. TALEN-transfected mIMCD3 clones were lysed (50 mM KCl, 1.5 mM MgCl_2_, 10 mM Tris pH 8.5, 0.45% Igepal CA-630, 0.45% Tween-20, 0.025% 20 mg/ml Proteinase K) for 2 h at 55 °C. Proteinase K (Roche) was then inactivated 10 min at 95 °C. Samples were subsequently genotyped (2x ReddyMix PCR Master Mix with 1.5 mM MgCl_2_; ThermoFischer Scientific) using the following oligonucleotides:

Pkd2-1: TTAGTAAGCGCGTGTCCA [43];
Pkd2-2: ACTGAGGCAAACTGCCAT [43];
Pkd2-3: TATGGCAGGTCCCTGTGG [43];
Slc25a25-1: GCTATCCAGAAGCCAAGC;
Slc25a25-2: CCACATGCCAACCTCAAC;
Slc25a25-3: TAAGGCCTTCAGCATCGT.

Wild-type PCRs for *Pkd2* and *Slc25a25* were performed with (1) and (2) and (4) and (5), respectively. Knockout alleles were detected by the combination of oligonucleotides (2) and (3) for *Pkd2* and (5) and (6) for *Slc25a25*. PCR products were sequenced to characterize genomic rearrangements (GATC-Biotech).

### RNA isolation and reverse transcription polymerase chain reaction (RT-PCR)

mRNA of mIMCD3 cells was isolated (RNeasy Plus Mini Kit, Qiagen) and retrotranscribed to complementary DNA (OneStepRT-PCR Kit, Qiagen) according to the manufacturer’s protocols using the following oligonucleotides:

36B4-1: CCGATCTGCAGACACACACT [67];
36b4-2: ACCCTGAAGTGCTCGACATC [67];
Pkd2-1: GTGGATGTACACAAGTGAGAAGGAGC [43];
Pkd2-2: CACGACAATCACAACATCCAGACA [43];
Slc25a25-1: AAACTAGTCCCGCGCTCGCTGTCTGA;
Slc25a25-2: AACTCGAGCGCAGGGACTGCATGATCTCCT.

### Ca^2+^ imaging

Fura-2-acetoxymethyl ester (Fura-2-AM)-based measurement of intracellular Ca^2+^ transients has been described previously [68,69]. Briefly, mIMCD3 cells were cultivated in 6 wells (Greiner Bio-One) on cover glasses (Carl Roth). Before measurement, confluent cells were cultivated for 48 h in DMEM F-12 (Lonza) without FBS. Cells were then washed twice with 2 ml HEPES Media (194 ml Ringer’s solution, 4 ml 1 M HEPES, 0.4 g D-glucose, 2 ml Penicilin/Streptomycin, pH 7.4) and incubated for 30 min at 37 °C in a humidified 10% CO_2_ incubator with 1 ml Fura-2-AM Loading Solution (4 μl Fura-2-AM, 2 μl 20% Pluronic F-127 in DMSO, 1 ml HEPES Media; ThermoFischer Scientific). To remove excess Fura-2-AM, cells were next washed twice with 2 ml HEPES Media, and incubation was continued for another 15 min. HEPES Media was replaced by DMEM F-12, and cover glasses were loaded into a parallel-plate flow chamber. One μM ATP (40 ml DMEM F-12, 2.2 mg ATP; Sigma-Aldrich) was used to induce intracellular Ca^2+^ transients. Life imaging was performed on an inverted Axio Observer microscope (Zeiss).

### Oxygen consumption rate (OCR)

OCR was measured using a Seahorse Biosciences XF^e^96 extracellular flux analyzer. Ten thousand cells per well were seeded in XF96 cell culture plates coated with poly-D-lysine (Sigma-Aldrich). Attachment of the cells was monitored after 1 h and 3 h, and cells were incubated over night at 37 °C with 5% CO_2_. Before the assay, cells were washed twice with XF base media (unbuffered DMEM supplemented with 2 mM L-Glutamine [Sigma-Aldrich], 11 mM Glucose [Carl Roth], 1 mM Sodium Pyruvate [Sigma-Aldrich] at pH 7.4) and equilibrated for 1 h at 37 °C without CO_2_. The OCR was measured afterwards using the following inhibitors: 2 μM oligomycin (Seahorse Biosciences), 1 μM carbonyl cyanide 4-(trifluoromethoxy)phenylhydrazone (FCCP, Seahorse Biosciences), and 2 μM rotenone with 2 μM antimycin A (Seahorse Biosciences). For each condition (basal and after each inhibitor injection), cycles were performed in triplicate with 3 min mixing followed by 3 min measurement. After completion of the assay, the protein content per well was determined using the Bradford assay (Roti-Quant; Carl Roth). Absorption was measured at 595 nm using a PerkinElmer 2030 Explorer plate reader. The OCR was normalized to the protein content for each well, and the results of inner wells of the 96-well plate with the same cell density were evaluated for the analysis of the cell lines.

### Nontargeted metabolomics

Nontargeted metabolomics have been described previously [70]. Briefly, mIMCD3 cells were cultivated in 6 wells (Greiner Bio-One) for 14 d (≥10 d 100% confluence). Cells were homogenized using 80 mg of 0.5 mm glass beads (Precellys) in 80% v/v methanolic solution. Sample was loaded in duplicates onto 96-well 350-μL PCR plates by splitting each into 2 aliquots consisting of 105 μl. The first aliquot was used for LC-MS/MS analysis in positive electrospray ionization mode, and the second aliquot was used for that in negative mode. Metabolites were annotated by curation of the LC-MS/MS data against proprietary Metabolon’s chemical database library (Metabolon) based on retention index, precursor mass, and MS/MS spectra. In this study, 199 known metabolites and 66 compounds with an unknown chemical structure, indicated by a letter X followed by a number as the compound identifier, were identified. The metabolites were assigned to cellular pathways based on PubChem, KEGG, and the Human Metabolome Database.

### Protein isolation, SDS-PAGE, western blot, and CCD camera-based ECL detection

Biochemical methods have been described previously [58,71].

### Immunofluorescence and super-resolution microscopy (stimulated emission depletion [STED])

Immunofluorescence of HeLa and mIMCD3 cells was described previously [58]. Briefly, cells were cultivated in 6 wells (Greiner Bio-One) on cover glasses (Carl Roth) until about 80% confluence. Primary antibody dilutions in PBS: anti-acetyl-α-tubulin 1:100; anti-Pyruvate dehydrogenase 1:1,000; anti-TRPP2 (G-20) 1:1,000; anti-V5 1:1,000. Mitochondria were visualized with pDsRed2-Mito (Clontech) according to the instructions of the manufacturer. A Leica TCS SP8 STED 3X microscope was used for confocal image acquisition. Vertical projections of recorded stacks were generated using ImageJ 2. STED was used to image close contacts between the ER and mitochondria. STED data were rendered in Imaris 8 (Bitplane). Brightfield images were recorded using an Axio Observer microscope (Zeiss).

### Flies

All flies were reared according to standard procedures and maintained at 25 °C. Wild-type *w*^*1118*^ and chromosomal deficiency *Df(3L)BSC380)* were obtained from the Bloomington Drosophila Stock Center [72]. *amo*^*1*^ mutant flies have been described [12,13]. *Z3-2147* flies were generated in a large-scale ethyl methanesulfonate mutagenesis [18]. Transgenic *w*^*1118*^ flies expressing *V5-SCaMC*, *V5-SCaMC*^*Δ1–268*^, *V5-SCaMC*^*EF*^, and *V5-SLC25A25* cDNA were generated by P-element insertion (The Bestgene).

### *D*. *melanogaster* fertility assays

*D*. *melanogaster* males of various genotypes were separated upon eclosion and maintained in isolation 2 d prior to mating with *w*^*1118*^ virgin females. For each test, 2 pairs of adults were allowed to mate for 5 d and then removed from the vial. After 10 d, the number of progeny that eclosed from each vial was counted. Fertility tests were evaluated using the Mann-Whitney U test. An asterisk indicates *P* ≤ 0.05.

### Dissection and immunofluorescence of *D*. *melanogaster* sperm

Dissection and preparation of testis and spermatozoa as well as the anti-Amo antiserum have been described [12,13]. Microscopy images were recorded using a Zeiss Axio Observer microscope (Zeiss).

### Zebrafish

Embryos were kept at 28.5 °C in E3 medium (5 mM NaCl, 0.17 mM KCl, 0.33 mM CaCl_2_, 0.33 MgSO_4_, 0.05% methylene blue) with 0.2 mM 1-Phenyl-2-thiourea (Sigma-Aldrich) to suppress pigmentation and staged according to somite number or hours post fertilization (hpf) [73]. The following strains were used: wild-type *AB/TL*; *Tg(wt1b*:*EGFP)* [74]; *Tg(actb2*:*Mmu*.*Arl13b-GFP)* [75]; and *pkd2*^*−/−*^ *cup*^*tc321*^ [25].

### Morpholino phosphorodiamidate antisense oligonucleotides (MOs)

Morpholino antisense oligonucleotides (Gene Tools) were designed to target the translation of the mRNA leading to a protein knockdown phenotype or to target an exon splice donor site causing splicing defects of the mRNA.

Standard Control: CCTCTTACCTCAGTTACAATTTATA;
p53^MO^: GCGCCATTGCTTTGCAAGAATTG [76];
pkd2^MO^: GATCAACCCGTTACCTGACAATACA [28];
slc25a23a^MO^: AGAGTTTATAACCAACTAACCATCA;
slc25a23b^MO^: AAAGGTTAATGCAGACCATCGTTGT
slc25a24^MO^: TATCTCTGGTGTGGAACTTACAGTT;
slc25a25a^MO^: AGTATTGCATCAAGATATACCTGCA;
slc25a25b^ATG^: AGCTCCTCTTGTGCATTTCACACGC;
slc25a25b^MO^: AAGCACGGTGTGGTTTTTGTTTACC.

MOs were diluted (in 100 mM KCl, 10 mM HEPES, 0.1% Phenol Red) and phenotypes were analyzed in a concentration-dependent fashion. A p53^MO^ was co-injected 1.5-fold with all MOs to attenuate possible off-target effects [76]. Injections were performed into 1-cell-stage embryos using a microinjector PLI-90 (Harvard Apparatus). Injection volume was approximately 2 nl comprising 8 ng pkd2^MO^, slc25a23a^MO^, slc25a23b^MO^, slc25a24^MO^, or slc25a25a^MO^; 5 ng slc25a25b^ATG^; 3 ng slc25a25b^E4-I34^; or Standard Control MO, respectively.

### Analysis of heart looping in zebrafish

Heart looping of live zebrafish embryos was analyzed blinded for genotype and injection 48 hpf. At least 3 independent experiments were evaluated using a Stereo Discovery V8 microscope (Zeiss).

### Rescue of MO-induced zebrafish phenotypes

The specificity of MOs was validated by mRNA-mediated rescue of respective phenotypes. Rescue experiments were done by co-injection of capped mRNA (mMESSAGE mMACHINE; ThermoFisher Scientific) and MOs. Transcripts used were *pkd2* (ENSDART00000020412) and *slc25a25b* (ENSDART00000098163).

### Riboprobes and whole-mount in situ hybridization

Whole-mount in situ hybridization was performed as described previously [77]. Digoxigenin-labeled RNA probes were transcribed from linearized DNA templates and used in RNA in situ hybridization. Stained embryos were transferred into glycerol and photographed with a Stereo Discovery V8 microscope (Zeiss). Riboprobes:

*cardiac myosin light chain 2* (*cmlc2*) [78];
*dand5* [79];
*lefty1* [80];
*lefty2* [80];
*pkd2* [25];
*preproinsulin* (*ins*) [81];
*slc25a25b* (ENSDART00000098163);
*southpaw* [82].

### Immunofluorescence and live imaging of *D*. *rerio* KV

Six-somite stage embryos *Tg(actb2*:*Mmu*.*Arl13b-GFP)* were embedded in 1% low-temperature melting agarose (Biozym) in 30% Danieau’s solution (17.4 mM NaCl, 0.21 mM KCl, 0.12 mM MgSO_4_ × 7 H_2_O, 0.18 mM Ca[NO_3_]_2_, 1.5 mM HEPES). Real-time imaging of GFP-labeled KV cilia was performed on a ZEISS LSM 510 Live confocal microscope equipped with a LD LCI Plan-Apochromat 25×/0.8 glycerine objective (Zeiss). Six hundred images were recorded per sample at 9–10 frames per second with a resolution of 512 × 512 pixels. Similarly, flow in the KV of embedded 8-somite stage embryos was visualized by particle tracking using differential interference contrast (DIC) microscopy (Zeiss Axio Observer microscope; Zeiss) for 2 min at 20 frames per second. [39]. For high-resolution 3D confocal imaging of zebrafish KV, *Tg(actb2*:*Mmu*.*Arl13b-GFP)* was fixed in methanol and stained anti-GFP to enhance contrast as described previously [83]. A Leica TCS SP8 STED 3X microscope was used for image acquisition. Vertical projections of recorded stacks were generated using ImageJ 2.

### Transmission electron microscopy

Electron microscopy of zebrafish embryos has been described previously [84]. Thin sections (approximately 70–80 nm) were cut on a Reichert Ultracut E ultramicrotome and collected onto formvar-coated slot grids. Sections were stained with uranyl acetate and lead. Samples were examined in a Philips CM10 TEM at 80 kV.

### Statistical analysis

Data presented are based on at least 3 independent experiments. Experiments were performed and analyzed blinded for the respective genotype. *D*. *melanogaster* fertility and *D*. *rerio* laterality were evaluated using the Mann-Whitney U test and the χ^2^ test, respectively. Metabolomics data were analyzed using Student *t* test with Bonferroni multiple significance test correction (statistical significance *P ≤ 0.00019). An asterisk indicates *P ≤ 0.05.

## Supporting information

S1 Fig Flies carrying the missense mutation CG32103^R308W^ phenocopied loss of TRPP2. (A) Schematic of the unbiased forward genetic screen of EMS mutant flies to identify CG32103^R308W^. (B) Sperm of wild-type male flies are transferred to the uterus after mating. (C) These sperm navigate into the seminal receptacle, a female sperm storage organ, a prerequisite for reproductive success. (D) *Z3-2147* flies produced motile sperm that were transferred to the uterus but (E) failed to reach the female sperm storage organs. (F) In mature wild-type sperm, Amo (TRPP2) clustered at the tip of the sperm tail. (G) Amo localization was not impaired in *Z3-2147* flies. Scale bars B,D = 50 μm, C,E = 20 μm, and F,G = 5 μm. EMS, ethyl methanesulfonate; TRPP2, transient receptor potential channel polycystin-2.
(TIF)

S2 Fig Expression of *SCaMC* (*CG32103*) cDNA constructs in fly sperm. Comparison of testis of wild-type males (A) to testis of transgenic males expressing V5-tagged cDNA constructs for (B) *SCaMC*, (C) *SCaMC*^*Δ1–268*^, and (D) *SCaMC*^*EF*^ (representative images, *n* ≥ 10). Expression levels of wild-type B and Ca^2+^ binding–deficient *SCaMC* transgenes C,D were similar. Scale bars = 100 μm.
(TIF)

S3 Fig SCaMC is a mitochondrial transport protein. (A) Schematic of SCaMC protein topology. (B) V5 tag–based immunofluorescence detection of *SCaMC* transgene expression in mature *D*. *melanogaster* sperm. (C) SCaMC expression mimicked the cellular distribution of the mitochondria-associated fusion protein dj^GFP^ along the entire sperm tail [85]. Scale bars B,C = 10 μm. dj^GFP^, Don Juan–green fluorescent protein.
(TIF)

S4 Fig Loss of *pkd2* expression in zebrafish caused randomization of left–right patterning. (A) In situ hybridization of *pkd2* mRNA in wild-type zebrafish 24 (B) and 48 hpf. (C,D) Left–right asymmetry was visualized by in situ hybridization for cmlc2 to evaluate heart looping. Knockdown of *pkd2* expression caused a randomization of heart looping [25]. cmlc2, cardiac myosin light chain 2; hpf, hours post fertilization.
(TIF)

S5 Fig Loss of *slc25a25b* expression in zebrafish caused randomization of left–right patterning. (A) SCaMC has 3 homologs in vertebrates SLC25A23, SLC25A24, and SLC25A25. In zebrafish, these proteins are encoded by *slc25a23a*, *slc25a23b*, *slc25a24*, *slc25a25a*, and *slc25a25b*. MO-induced knockdown of *slc25a25b* caused randomized heart looping in zebrafish (*slc25a25b*^*MO*^; *P = 1.1 × 10^−34^). (B) Similar to splice-blocking *slc25a25b*^MO^, translation-blocking *slc25a25b*^ATG^ MOs induced randomization of heart looping in zebrafish embryos (*P = 3.2 × 10^−13^). Similar to *D*. *melanogaster* (Fig 2E), this phenotype was rescued by injection (+) of human SLC25A25 mRNA in a concentration-dependent fashion (*+SLC25A25*). (C) In situ hybridization of *slc25a25b* mRNA in wild-type zebrafish 24 hpf, (D) 48 hpf, and (E) at 10-somite stage. (F) Lateral pancreas placement—visualized by in situ hybridization of *preproinsulin* (*ins*)—was altered in *slc25a25b* morphants, highlighting a general heterotaxy defect 52 hpf (*n* = 28; left = 10; center = 14; right = 4; in comparison to Control^MO^: *n* = 24; left = 2; center = 10; right = 12; *P = 0.002). Numbers of embryos are indicated above bars. L = left; S = symmetric; R = right. For numerical values, see S1 Data. hpf, hours post fertilization; MO, Morpholino-oligonucleotide; SLC25A25, solute carrier 25 A 25.
(TIF)

S6 Fig Kupffer’s vesicle morphology in control and slc25a25-morphant fish. Number and overall morphology of cilia in zebrafish Kupffer’s vesicle appeared normal in (A) control and (B) *slc25a25b*-morphant embryos, as visualized in transgenic arl13b^GFP^ fish (representative images, *n* ≥ 20). Scale bars = 10 μm. slc25a25, solute carrier 25 A 25.
(TIF)

S7 Fig Structure and motility of cilia in zebrafish Kupffer’s vesicle appeared normal at the 8-somite stage. (A) Still image of S2 Movie from Kupffer’s vesicle of *pkd2*-morphant embryos shows cilia in 1 focal plane. It has been shown previously that knockdown of *pkd2* does not affect cilia number and motility [25]. (B) *slc25a25b*-morphant Kupffer’s vesicle cilia resembled *pkd2*-morphant cilia in (C) number, (D) beating, and (E) length. Numbers of cilia are indicated in bars. *pkd2*^MO^: *n* = 20; mean number of cilia / Kupffer’s vesicle = 25.05 (standard deviation = 8.69); percentage of beating cilia = 44.525 (standard deviation = 12.55); average length of cilia = 5.05 μm (standard deviation = 1.33). *slc25a25b*^MO^: *n* = 21; mean number of cilia / Kupffer’s vesicle = 17.32 (standard deviation = 8.2); percentage of beating cilia = 40.154 (standard deviation = 17.5); average length of cilia = 5.77 μm (standard deviation = 1.61). Similar to (F) wild type, (G) *pkd2*^*MO*^ and (H) *slc25a25b*^*MO*^ fish generate effective directional flow in the Kupffer’s vesicle as visualized by particle tracking at the 8-somite stage [86,87]. No significant differences were observed in flow velocities (wild type: *n* = 6; mean velocity = 10.2 μm/s; standard deviation = 2.4; *pkd2*^*MO*^: *n* = 9; mean velocity = 10.4 μm/s; standard deviation = 2.2; *slc25a25b*^*MO*^: *n* = 10; mean velocity = 7.9 μm/s; standard deviation = 2.2). Scale bars = 20 μm. For numerical values, see S1 Data.
(TIF)

S8 Fig *slc25a25b* acts upstream of the *southpaw* (*nodal*) cascade. (A-C) *southpaw* expression in *pkd2*-morphant zebrafish embryos. Loss of *pkd2* caused left–right randomization of *southpaw* expression. *southpaw* mRNA in 15-somite stage zebrafish embryos was visualized by in situ hybridization. (D) Comparison of *southpaw* expression patterns in control and *pkd2*-morphant (*P = 0.0005) zebrafish. (E-G) Expression of *dand5* in 6-somite stage *slc25a25b*-morphant zebrafish embryos. Asymmetry of *dand5* expression was impaired in 6-somite *slc25a25b*-morphants (wild type: *n* = 27; left = 4; symmetric = 11; right = 12; Control^MO^: *n* = 28; left = 5; symmetric = 12; right = 11; slc25a25b^MO^: *n* = 18; left = 10; symmetric = 5; right = 3; in comparison to Control^MO^ *P = 0.03) as well as in (H) 8-somite *slc25a25b*-morphants (*P = 0.007). (I,J) *lefty2* expression in slc25a25b-morphant zebrafish embryos 22 hpf. (K) *lefty2* expression was randomized in *pkd2*- and *slc25a25b*-morphant zebrafish (*P = 2.2 × 10^−12^ and *P = 3.4 × 10^−8^, respectively). Numbers of embryos are indicated above bars. L = left; S = symmetric; R = right. For numerical values, see S1 Data. hpf, hours post fertilization.
(TIF)

S9 Fig Proximity of mitochondria and cilia. (A) Similar to Kupffer’s vesicle, cilia and mitochondria localize closely at the apical pole of in epithelial cells. Representative confocal, apical, 0.22 μm sections of mIMCD3 cells. Primary cilia are stained using an anti-acetylated tubulin (“aTub”) antibody. Mitochondria are visualized using anti-pyruvate dehydrogenase antibody (“PD”). Scale bar = 4 μm. (B) 2D histogram of the fluorescence intensities in Fig 5C (single slice, overlay of green and magenta channel after deconvolution of the individual 3D stacks). Colocalization would be indicated by a cluster of signals at the xy diagonal line. In line with the close proximity but not colocalization of the TRPP2- and SLC25A25-expressing organelles in the analyzed HeLa cells—ER and mitochondria—the widely scattered signals in the histogram indicate a limited degree of colocalization. ER, endoplasmic reticulum, SLC25A25, solute carrier 25 A 25; TRPP2, transient receptor potential channel polycystin-2.
(TIF)

S10 Fig TALEN-mediated deletion of *Pkd2* (TRPP2) and *Slc25a25* in mIMCD3 cells. (A) Genomic structure of mIMCD3 *Pkd2*^*−/−*^ cells [43]. (B) *Pkd2* RT-PCR. (C) Immunoprecipitation of TRPP2. (D) Genomic structure of mIMCD3 *Slc25a25*^*−/−*^ cells. (E) *Slc25a25* RT-PCR. RT-PCR, reverse transcription polymerase chain reaction; TALEN, transcription activator-like effector nuclease; TRPP2, transient receptor potential channel polycystin-2.
(TIF)

S11 Fig Functional characterization of mutant mIMCD3 cells. (A) Fura-2-AM-based intracellular Ca^2+^ measurement of ATP-induced (1 μM) Ca^2+^ transients in wt and *Slc25a25*^*−/−*^ mIMCD3 cells. (B) No significant differences were observed (*n* = 10). (C) Oxygen consumption rates were measured at basal conditions and after injection of oligomycin (I.), FCCP (II.), and antimycin A plus rotenone (III.) at the indicated time points. The results are presented as mean ± standard error of the mean (*n* = 6). For numerical values see S1 Data. FCCP, carbonyl cyanide 4-(trifluoromethoxy)phenylhydrazone; Fura-2-AM, Fura-2-acetoxymethyl ester; wt, wild-type.
(TIF)

S12 Fig Combined knockdown of *pkd2* and *slc25a25b*. Individual application of *slc25a25b*^MO^ and *pkd2*^MO^ caused a significant increase in heart looping defects (*P = 0.01 and *P = 7 × 10^−7^, respectively). Parallel knockdown of *slc25a25b* and *pkd2* did not aggravate the phenotype. Injection volume was approximately 2 nl comprising 5 ng Control^MO^, 1.5 ng *slc25a25b*^MO^ + 2.5 ng Control^MO^, 2.5 ng *pkd2*^MO^ + 1.5 ng Control^MO^, or 2.5 ng *pkd2*^MO^ + 1.5 ng *slc25a25b*^MO^. For numerical values, see S1 Data.
(TIF)

S1 Table Changed metabolites observed for *Pkd2*^*−/−*^ and *Slc25a25*^*−/−*^ genotypes. (DOCX)

S1 Data Numerical values used to generate summary figures. (XLSX)

S2 Data Metabolites were annotated by curation of the LC-MS/MS data against proprietary Metabolon’s chemical database library based on retention index, precursor mass, and MS/MS spectra. In this study, 199 known metabolites and 66 compounds with an unknown chemical structure, indicated by a letter X followed by a number as the compound identifier, were identified. The metabolites were assigned to cellular pathways based on PubChem, KEGG, and the Human Metabolome Database. KEGG, Kyoto Encyclopedia of Genes and Genomes; LC-MS/MS, liquid chromatography–tandem mass spectrometry.
(XLSX)

S1 Movie Live imaging of sperm in the seminal receptacle of the female. The sperm expressed a dj^GFP^ protein but are otherwise wild type (9 frames/s; real time). Scale bar = 20 μm. dj^GFP^, Don Juan–green fluorescent protein.
(MOV)

S2 Movie Live imaging of zebrafish Kupffer’s vesicle cilia at the 8-somite stage. Cilia were evaluated using confocal microscopy. Sixty s were recorded from the focal plane with most cilia (9 frames/s; real time). Scale bar = 20 μm.
(MOV)

## Abbreviations

APC: ATP-Mg/P_i_ carrier
BCAA: branched-chain amino acid
*cmlc2*: *cardiac myosin light chain 2*
CPM: N-[4-(7-diethylamino-4-methyl-3-coumarinyl)-phenyl]-maleimide
DIC: differential interference contrast
ER: endoplasmic reticulum
FBS: fetal bovine serum
FCCP: carbonyl cyanide 4-(trifluoromethoxy)phenylhydrazone
Fura-2-AM: Fura-2-acetoxymethyl ester
hpf: hours post fertilization
HRM: high-resolution melt
KV: Kupffer’s vesicle
MO: morpholino phosphorodiamidate antisense oligonucleotide
OCR: oxygen consumption rate
RT-PCR: reverse transcription polymerase chain reaction
SC-Trp: synthetic complete tryptophan dropout
SLC25A25: solute carrier 25 A 25
STED: stimulated emission depletion
TALEN: transcription activator-like effector nuclease
TRPP2: transient receptor potential channel polycystin-2
