# Supplementary figures and images for: The mitochondrial transporter SLC25A25 links ciliary TRPP2 signaling and cellular metabolism
Source: PLoS Biol. 2018 Aug 6;16(8):e2005651. doi: 10.1371/journal.pbio.2005651 (PMC6095617; doi:10.1371/journal.pbio.2005651)

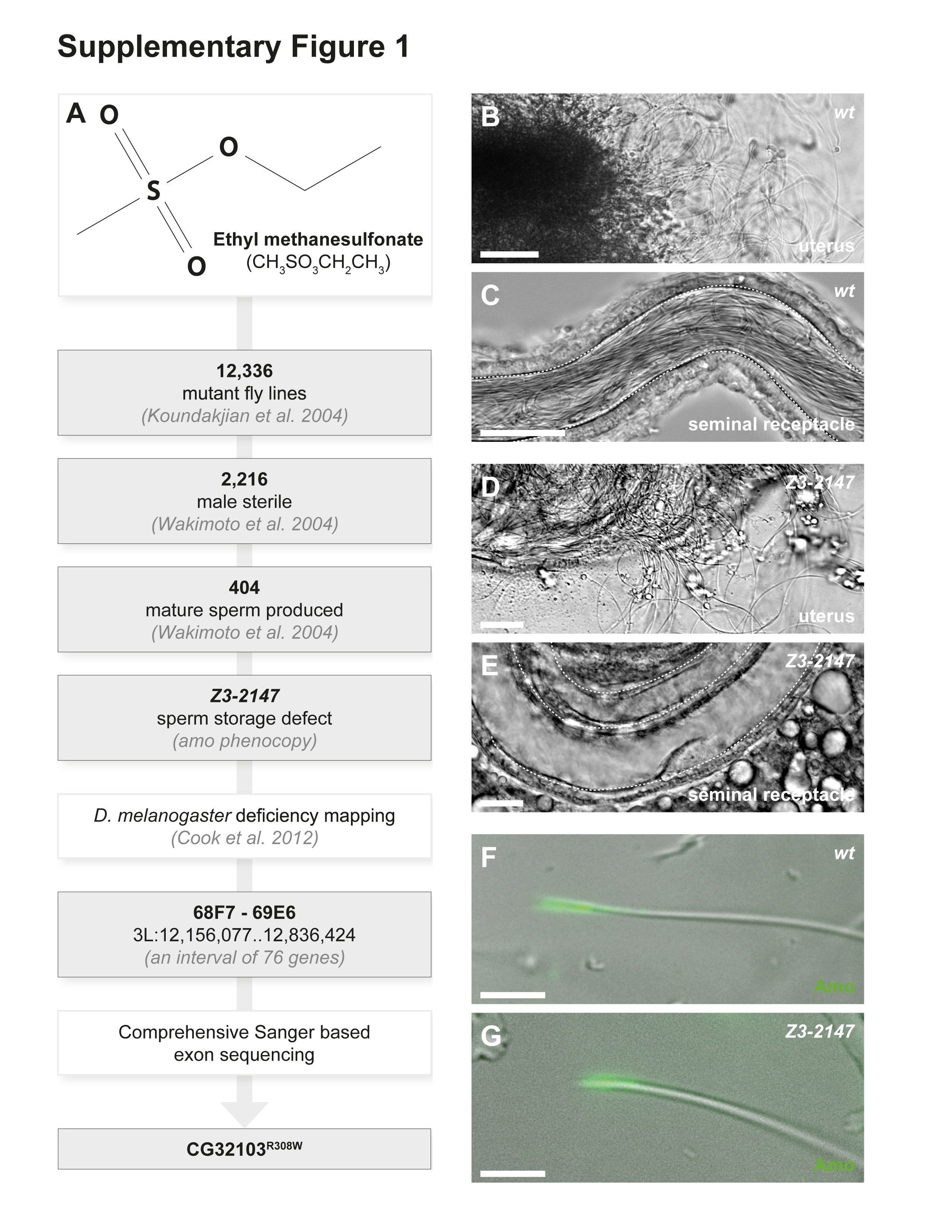

Supplement: S1 Fig — (A) Schematic of the unbiased forward genetic screen of EMS mutant flies to identify CG32103R308W. (B) Sperm of wild-type male flies are transferred to the uterus after mating. (C) These sperm navigate into the seminal receptacle, a female sperm storage organ, a prerequisite for reproductive success. (D) Z3-2147 flies produced motile sperm that were transferred to the uterus but (E) failed to reach the female sperm storage organs. (F) In mature wild-type sperm, Amo (TRPP2) clustered at the tip of the sperm tail. (G) Amo localization was not impaired in Z3-2147 flies. Scale bars B,D = 50 μm, C,E = 20 μm, and F,G = 5 μm. EMS, ethyl methanesulfonate; TRPP2, transient receptor potential channel polycystin-2. (TIF) [file pbio.2005651.s001.tif]

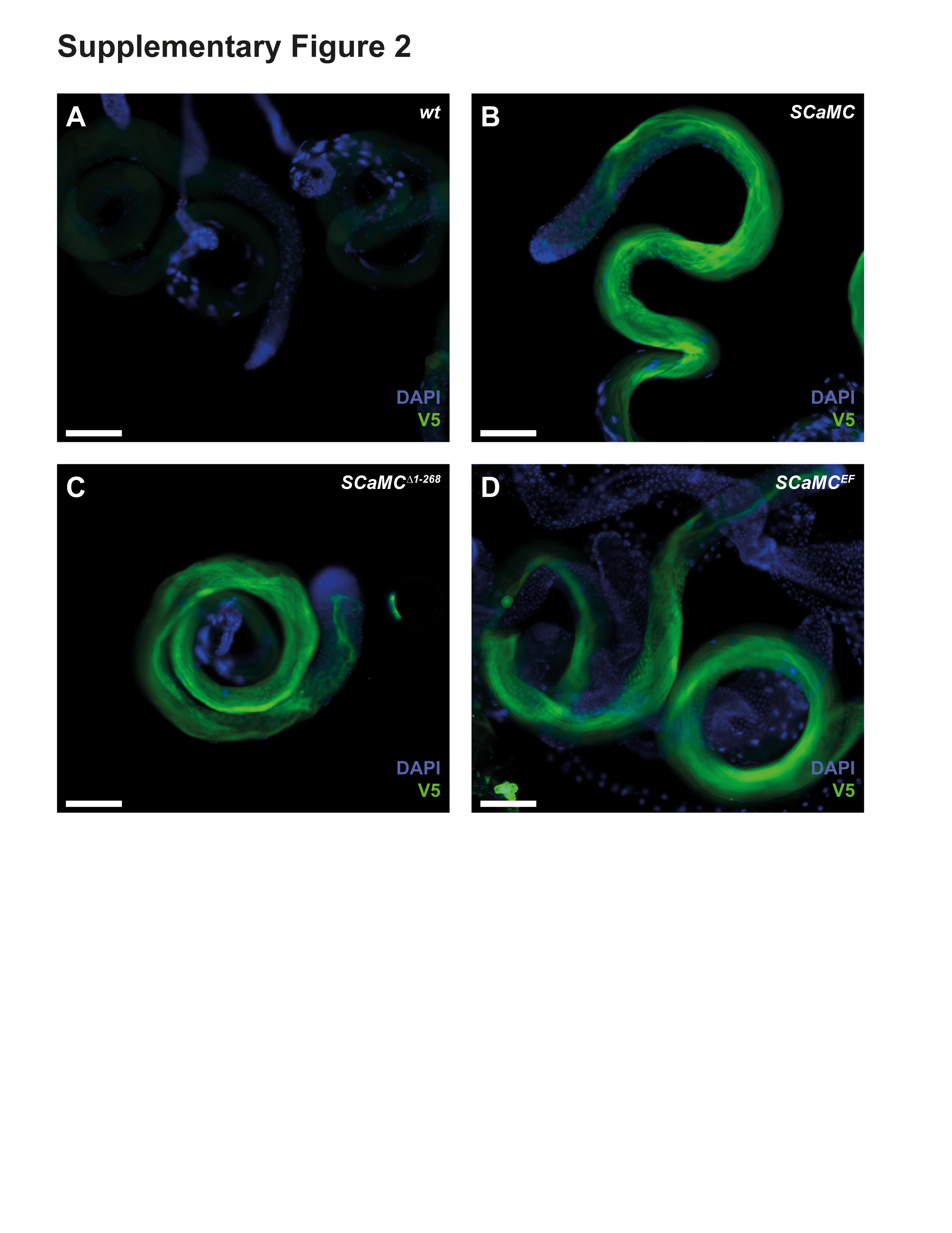

Supplement: S2 Fig — Comparison of testis of wild-type males (A) to testis of transgenic males expressing V5-tagged cDNA constructs for (B) SCaMC, (C) SCaMCΔ1–268, and (D) SCaMCEF (representative images, n ≥ 10). Expression levels of wild-type B and Ca2+ binding–deficient SCaMC transgenes C,D were similar. Scale bars = 100 μm. (TIF) [file pbio.2005651.s002.tif]

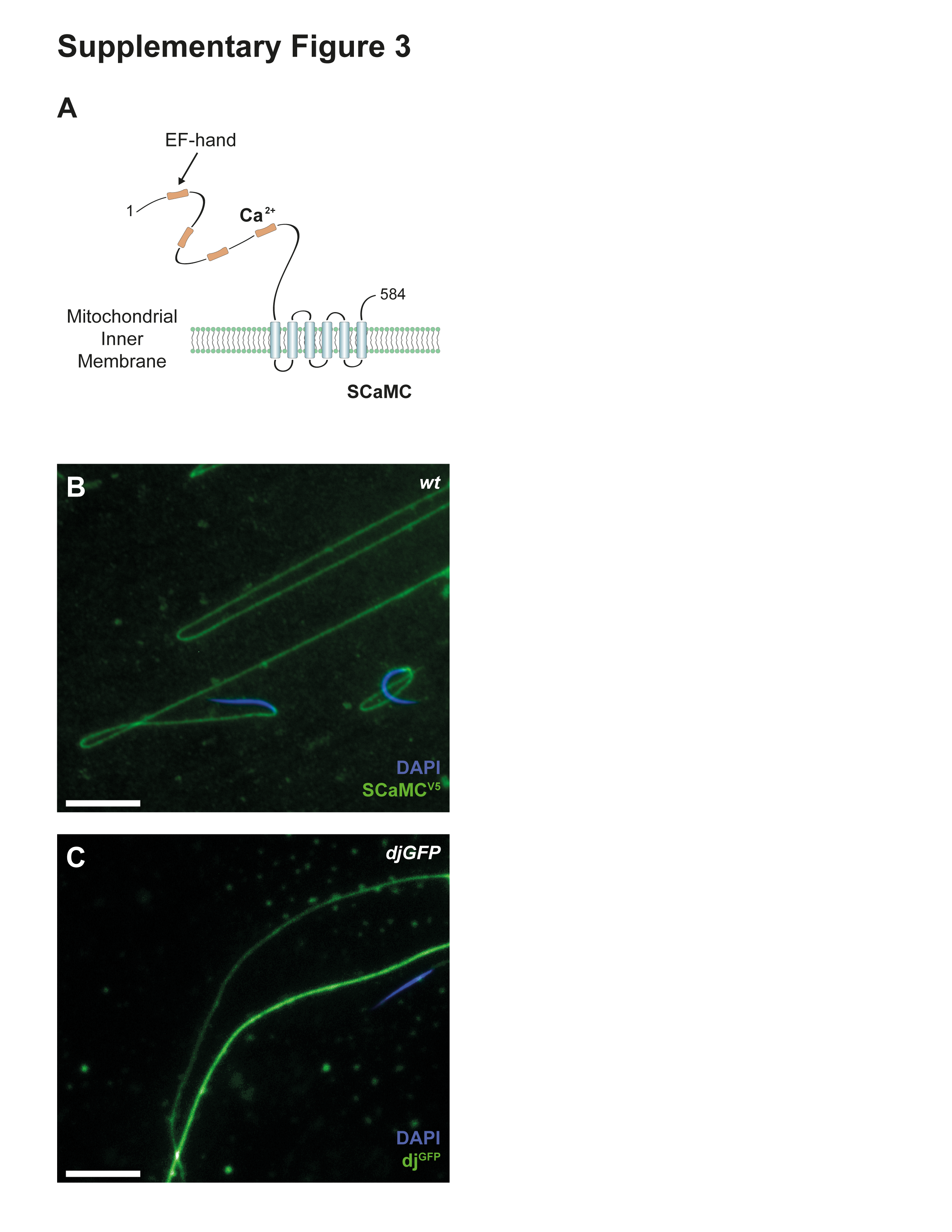

Supplement: S3 Fig — (A) Schematic of SCaMC protein topology. (B) V5 tag–based immunofluorescence detection of SCaMC transgene expression in mature D. melanogaster sperm. (C) SCaMC expression mimicked the cellular distribution of the mitochondria-associated fusion protein djGFP along the entire sperm tail [85]. Scale bars B,C = 10 μm. djGFP, Don Juan–green fluorescent protein. (TIF) [file pbio.2005651.s003.tif]

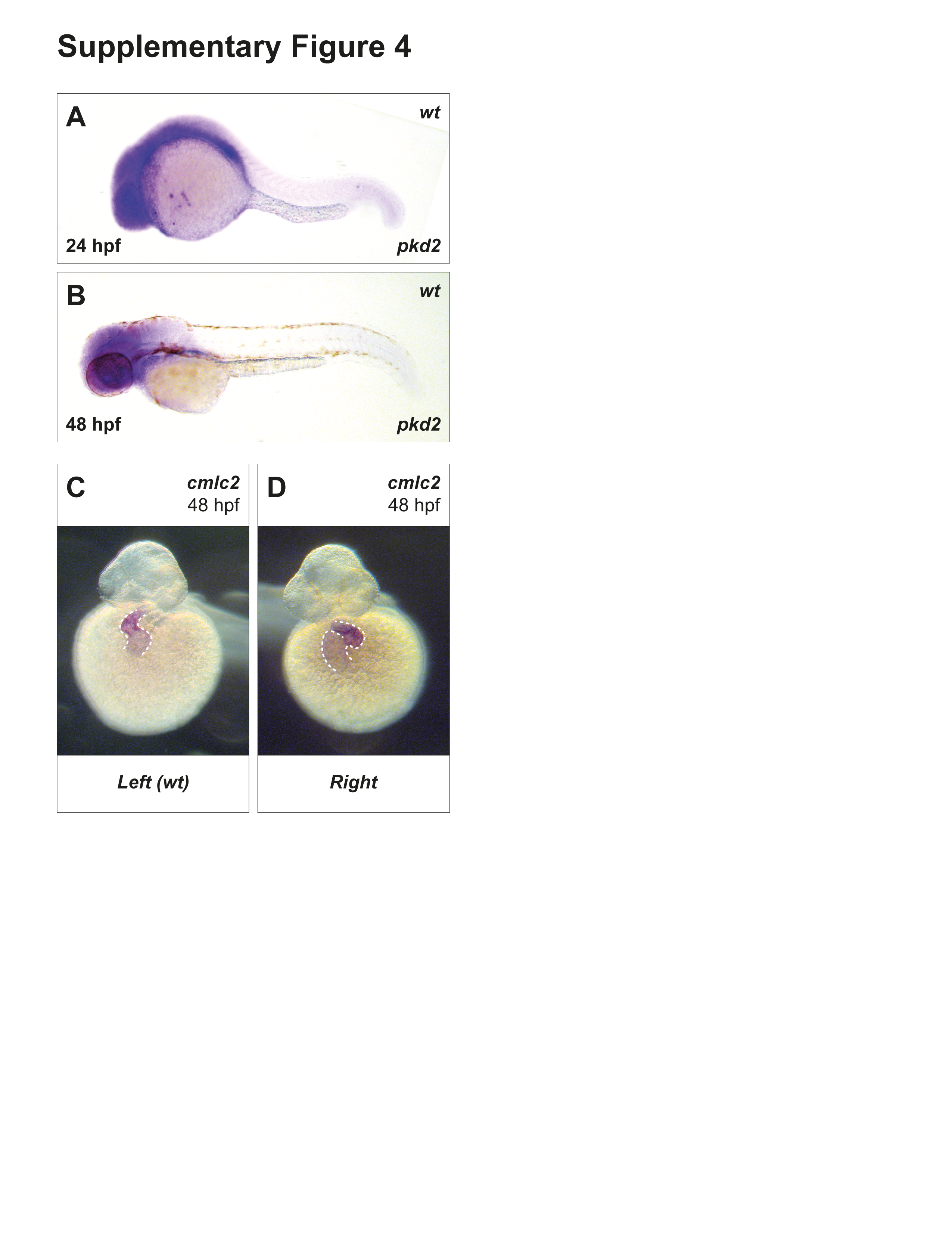

Supplement: S4 Fig — (A) In situ hybridization of pkd2 mRNA in wild-type zebrafish 24 (B) and 48 hpf. (C,D) Left–right asymmetry was visualized by in situ hybridization for cmlc2 to evaluate heart looping. Knockdown of pkd2 expression caused a randomization of heart looping [25]. cmlc2, cardiac myosin light chain 2; hpf, hours post fertilization. (TIF) [file pbio.2005651.s004.tif]

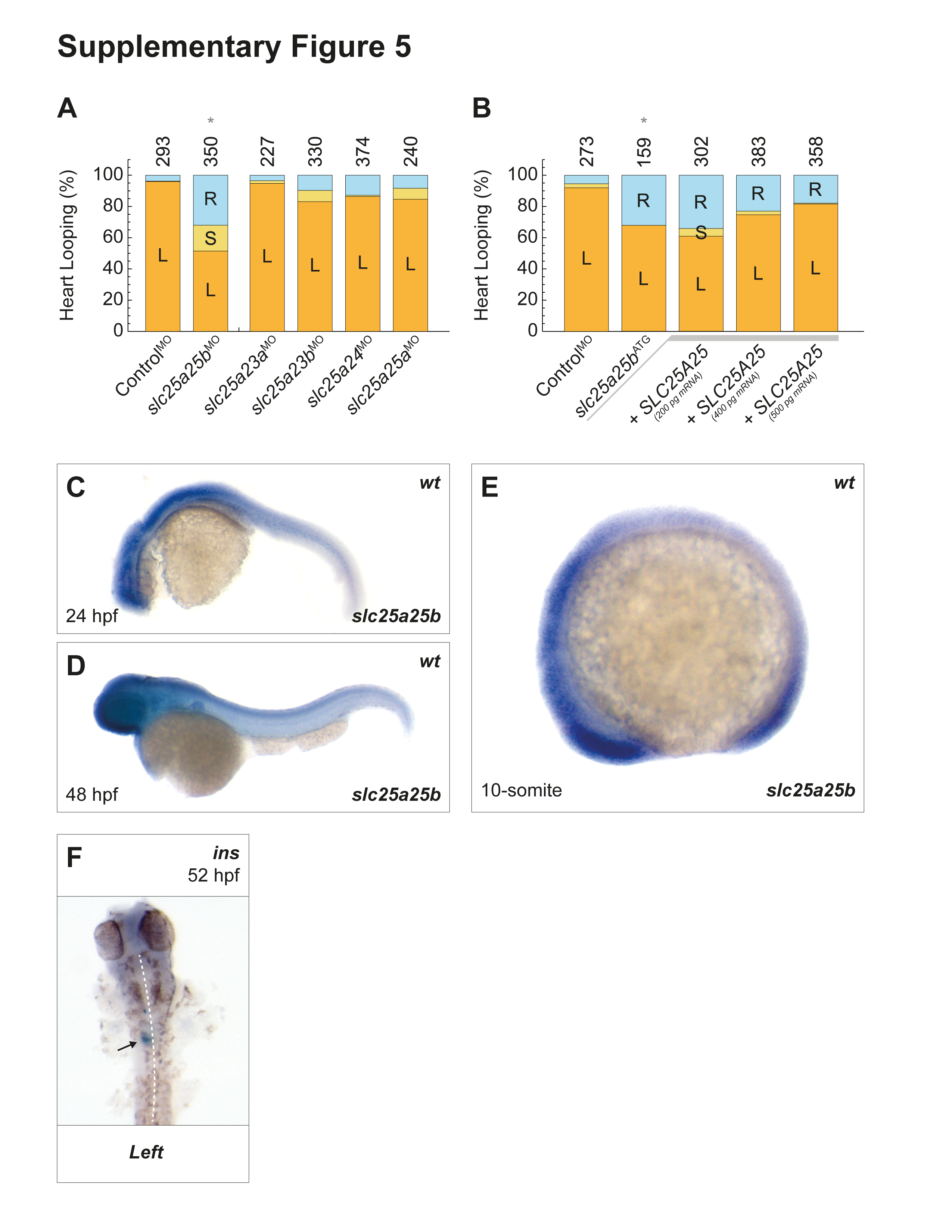

Supplement: S5 Fig — (A) SCaMC has 3 homologs in vertebrates SLC25A23, SLC25A24, and SLC25A25. In zebrafish, these proteins are encoded by slc25a23a, slc25a23b, slc25a24, slc25a25a, and slc25a25b. MO-induced knockdown of slc25a25b caused randomized heart looping in zebrafish (slc25a25bMO; *P = 1.1 × 10^−34^). (B) Similar to splice-blocking slc25a25bMO, translation-blocking slc25a25bATG MOs induced randomization of heart looping in zebrafish embryos (*P = 3.2 × 10^−13^). Similar to D. melanogaster (Fig 2E), this phenotype was rescued by injection (+) of human SLC25A25 mRNA in a concentration-dependent fashion (+SLC25A25). (C) In situ hybridization of slc25a25b mRNA in wild-type zebrafish 24 hpf, (D) 48 hpf, and (E) at 10-somite stage. (F) Lateral pancreas placement—visualized by in situ hybridization of preproinsulin (ins)—was altered in slc25a25b morphants, highlighting a general heterotaxy defect 52 hpf (n = 28; left = 10; center = 14; right = 4; in comparison to ControlMO: n = 24; left = 2; center = 10; right = 12; *P = 0.002). Numbers of embryos are indicated above bars. L = left; S = symmetric; R = right. For numerical values, see S1 Data. hpf, hours post fertilization; MO, Morpholino-oligonucleotide; SLC25A25, solute carrier 25 A 25. (TIF) [file pbio.2005651.s005.tif]

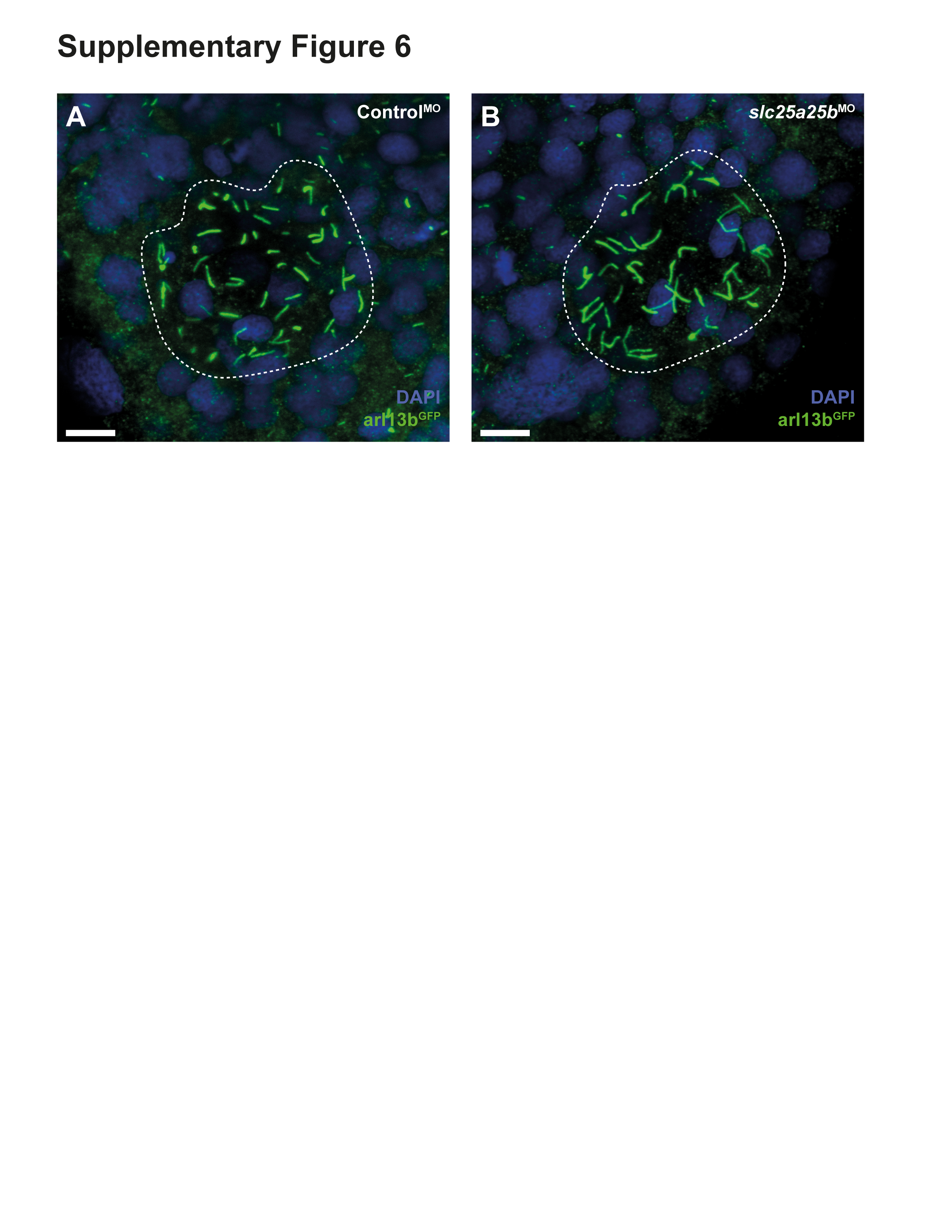

Supplement: S6 Fig — Number and overall morphology of cilia in zebrafish Kupffer’s vesicle appeared normal in (A) control and (B) slc25a25b-morphant embryos, as visualized in transgenic arl13bGFP fish (representative images, n ≥ 20). Scale bars = 10 μm. slc25a25, solute carrier 25 A 25. (TIF) [file pbio.2005651.s006.tif]

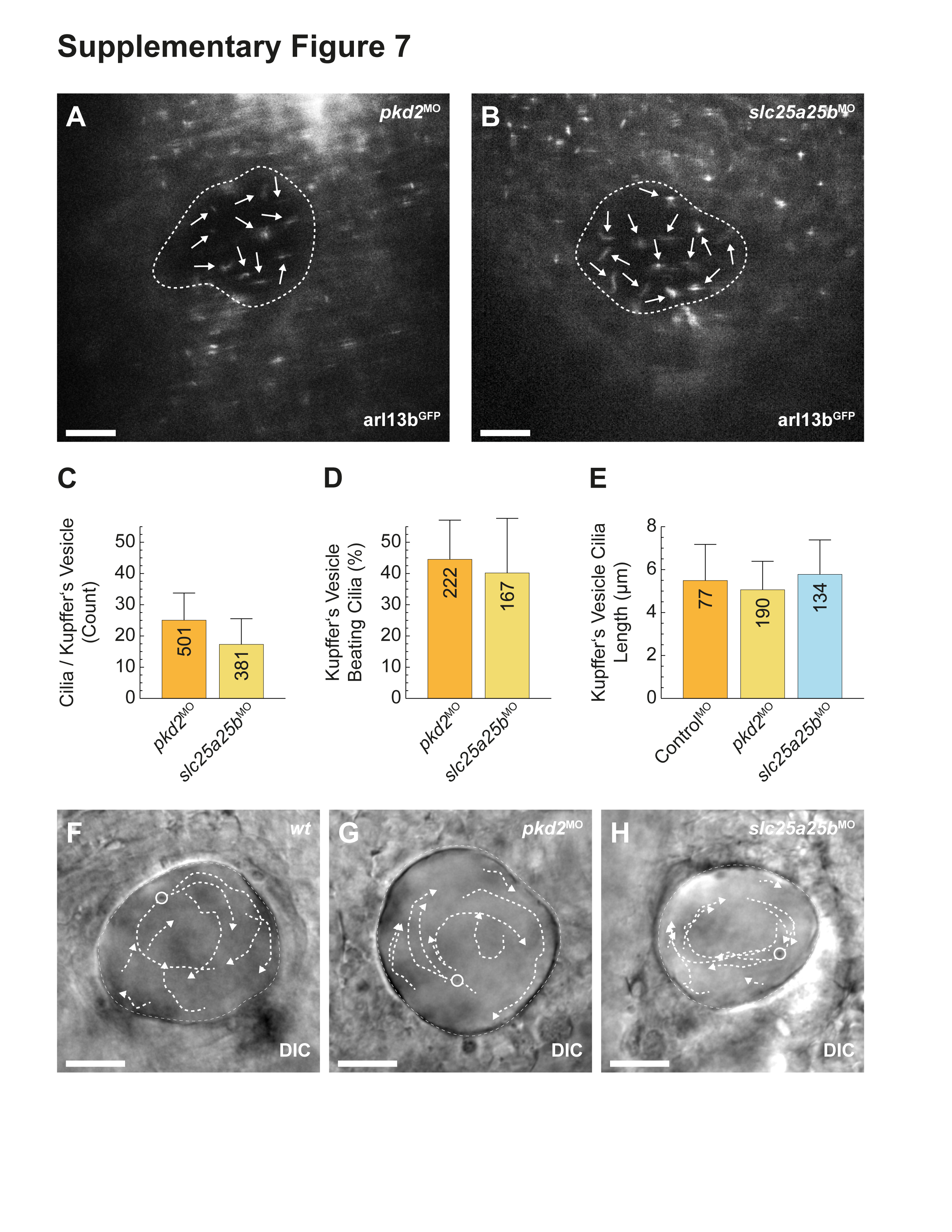

Supplement: S7 Fig — (A) Still image of S2 Movie from Kupffer’s vesicle of pkd2-morphant embryos shows cilia in 1 focal plane. It has been shown previously that knockdown of pkd2 does not affect cilia number and motility [25]. (B) slc25a25b-morphant Kupffer’s vesicle cilia resembled pkd2-morphant cilia in (C) number, (D) beating, and (E) length. Numbers of cilia are indicated in bars. pkd2MO: n = 20; mean number of cilia / Kupffer’s vesicle = 25.05 (standard deviation = 8.69); percentage of beating cilia = 44.525 (standard deviation = 12.55); average length of cilia = 5.05 μm (standard deviation = 1.33). slc25a25bMO: n = 21; mean number of cilia / Kupffer’s vesicle = 17.32 (standard deviation = 8.2); percentage of beating cilia = 40.154 (standard deviation = 17.5); average length of cilia = 5.77 μm (standard deviation = 1.61). Similar to (F) wild type, (G) pkd2MO and (H) slc25a25bMO fish generate effective directional flow in the Kupffer’s vesicle as visualized by particle tracking at the 8-somite stage [86,87]. No significant differences were observed in flow velocities (wild type: n = 6; mean velocity = 10.2 μm/s; standard deviation = 2.4; pkd2MO: n = 9; mean velocity = 10.4 μm/s; standard deviation = 2.2; slc25a25bMO: n = 10; mean velocity = 7.9 μm/s; standard deviation = 2.2). Scale bars = 20 μm. For numerical values, see S1 Data. (TIF) [file pbio.2005651.s007.tif]

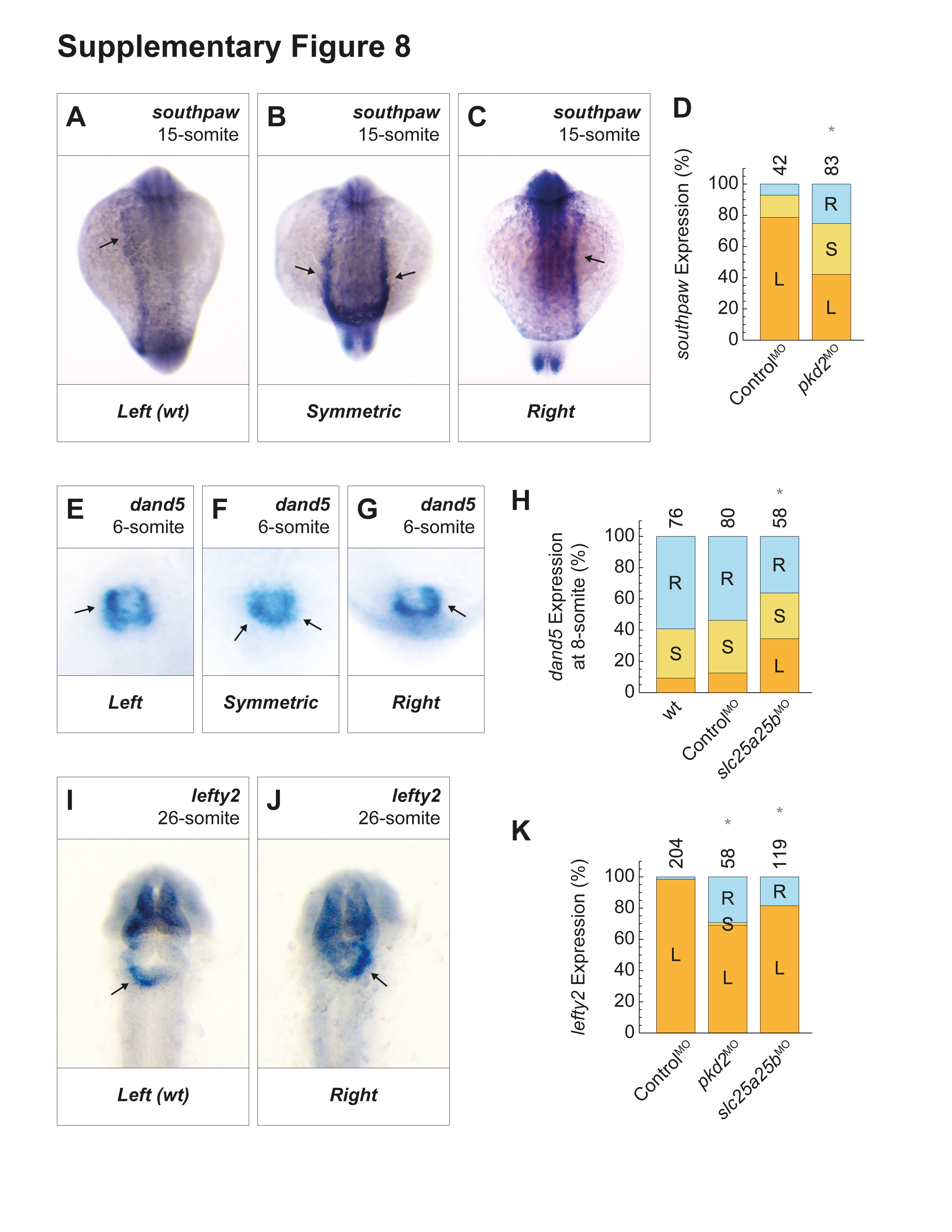

Supplement: S8 Fig — (A-C) southpaw expression in pkd2-morphant zebrafish embryos. Loss of pkd2 caused left–right randomization of southpaw expression. southpaw mRNA in 15-somite stage zebrafish embryos was visualized by in situ hybridization. (D) Comparison of southpaw expression patterns in control and pkd2-morphant (*P = 0.0005) zebrafish. (E-G) Expression of dand5 in 6-somite stage slc25a25b-morphant zebrafish embryos. Asymmetry of dand5 expression was impaired in 6-somite slc25a25b-morphants (wild type: n = 27; left = 4; symmetric = 11; right = 12; ControlMO: n = 28; left = 5; symmetric = 12; right = 11; slc25a25bMO: n = 18; left = 10; symmetric = 5; right = 3; in comparison to ControlMO *P = 0.03) as well as in (H) 8-somite slc25a25b-morphants (*P = 0.007). (I,J) lefty2 expression in slc25a25b-morphant zebrafish embryos 22 hpf. (K) lefty2 expression was randomized in pkd2- and slc25a25b-morphant zebrafish (*P = 2.2 × 10^−12^ and *P = 3.4 × 10^−8^, respectively). Numbers of embryos are indicated above bars. L = left; S = symmetric; R = right. For numerical values, see S1 Data. hpf, hours post fertilization. (TIF) [file pbio.2005651.s008.tif]

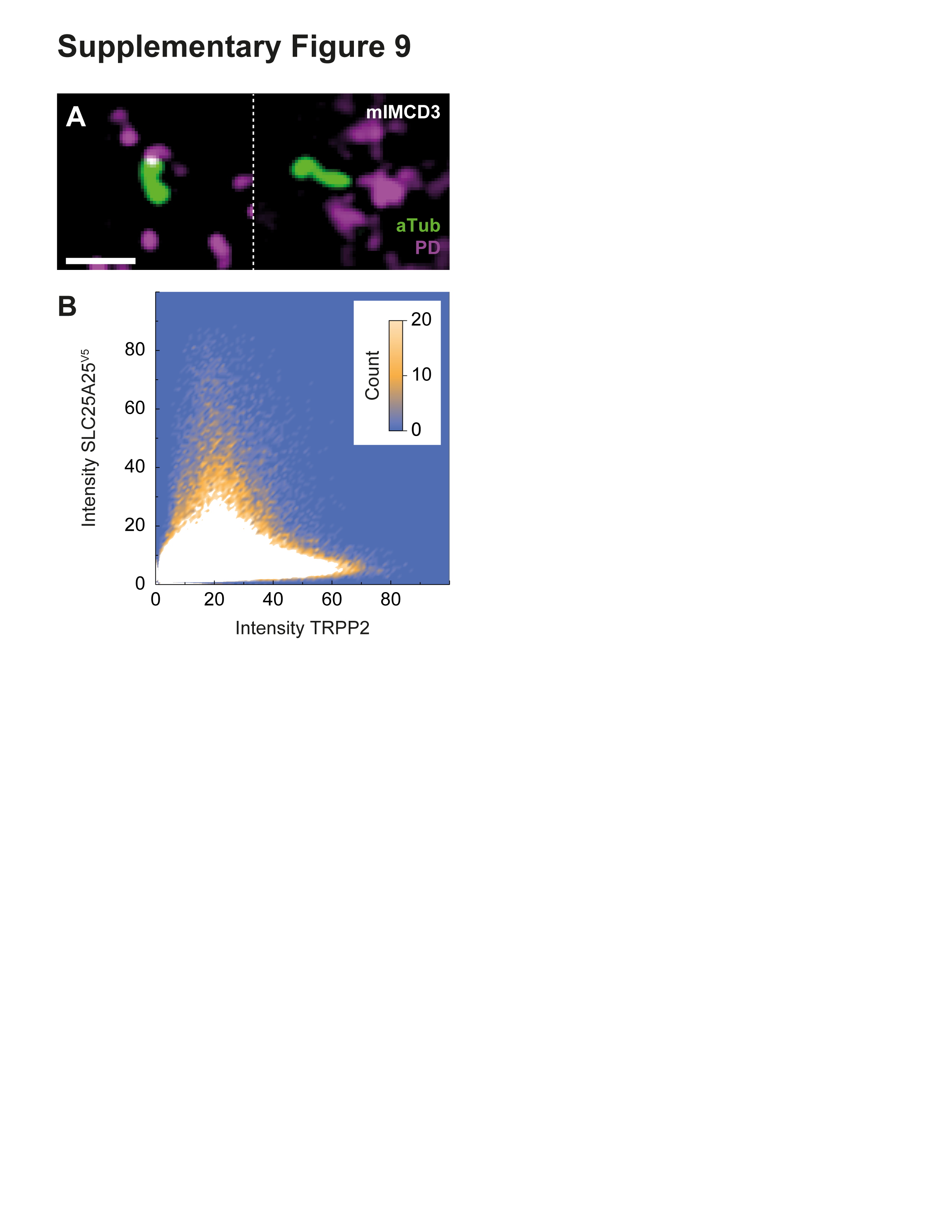

Supplement: S9 Fig — (A) Similar to Kupffer’s vesicle, cilia and mitochondria localize closely at the apical pole of in epithelial cells. Representative confocal, apical, 0.22 μm sections of mIMCD3 cells. Primary cilia are stained using an anti-acetylated tubulin (“aTub”) antibody. Mitochondria are visualized using anti-pyruvate dehydrogenase antibody (“PD”). Scale bar = 4 μm. (B) 2D histogram of the fluorescence intensities in Fig 5C (single slice, overlay of green and magenta channel after deconvolution of the individual 3D stacks). Colocalization would be indicated by a cluster of signals at the xy diagonal line. In line with the close proximity but not colocalization of the TRPP2- and SLC25A25-expressing organelles in the analyzed HeLa cells—ER and mitochondria—the widely scattered signals in the histogram indicate a limited degree of colocalization. ER, endoplasmic reticulum, SLC25A25, solute carrier 25 A 25; TRPP2, transient receptor potential channel polycystin-2. (TIF) [file pbio.2005651.s009.tif]

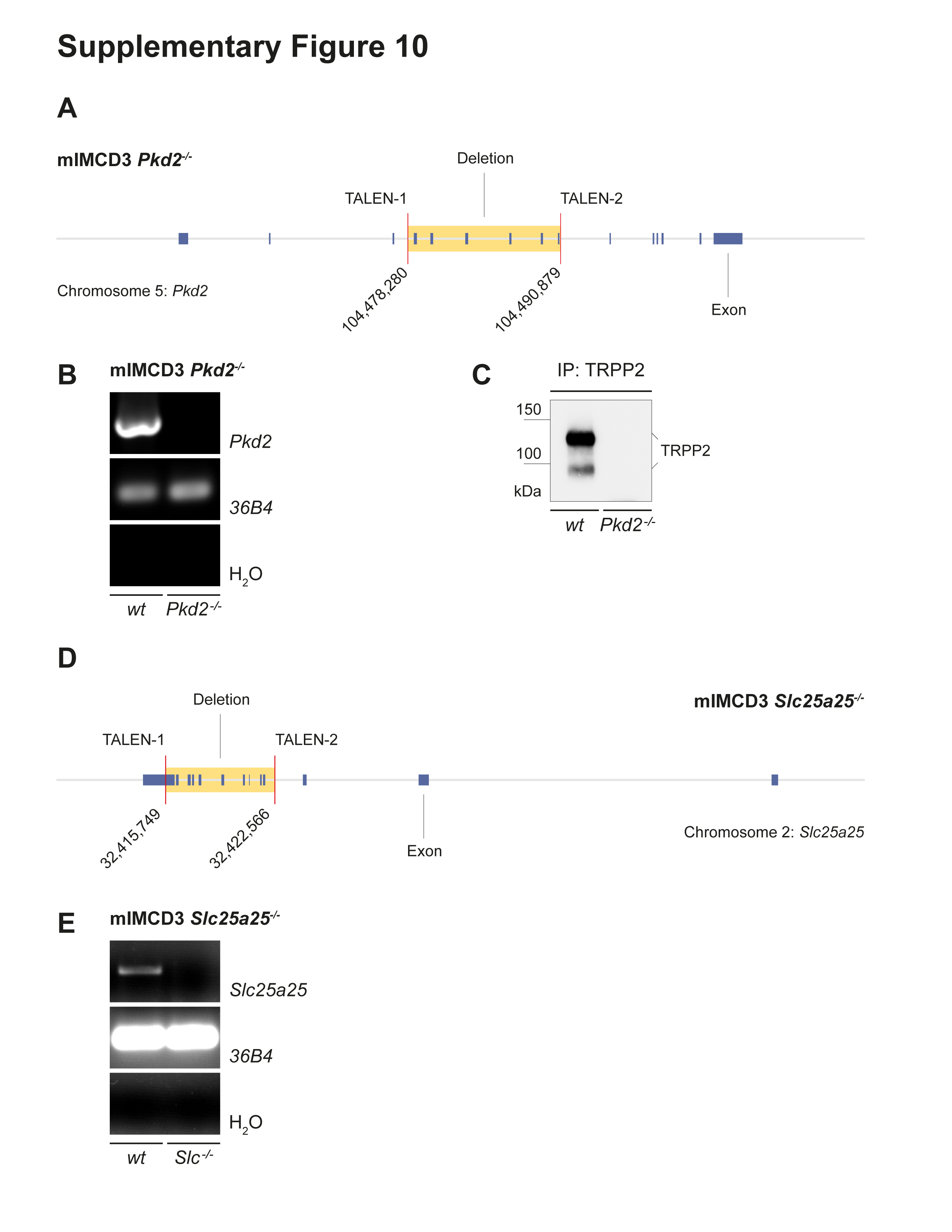

Supplement: S10 Fig — (A) Genomic structure of mIMCD3 Pkd2−/− cells [43]. (B) Pkd2 RT-PCR. (C) Immunoprecipitation of TRPP2. (D) Genomic structure of mIMCD3 Slc25a25−/− cells. (E) Slc25a25 RT-PCR. RT-PCR, reverse transcription polymerase chain reaction; TALEN, transcription activator-like effector nuclease; TRPP2, transient receptor potential channel polycystin-2. (TIF) [file pbio.2005651.s010.tif]

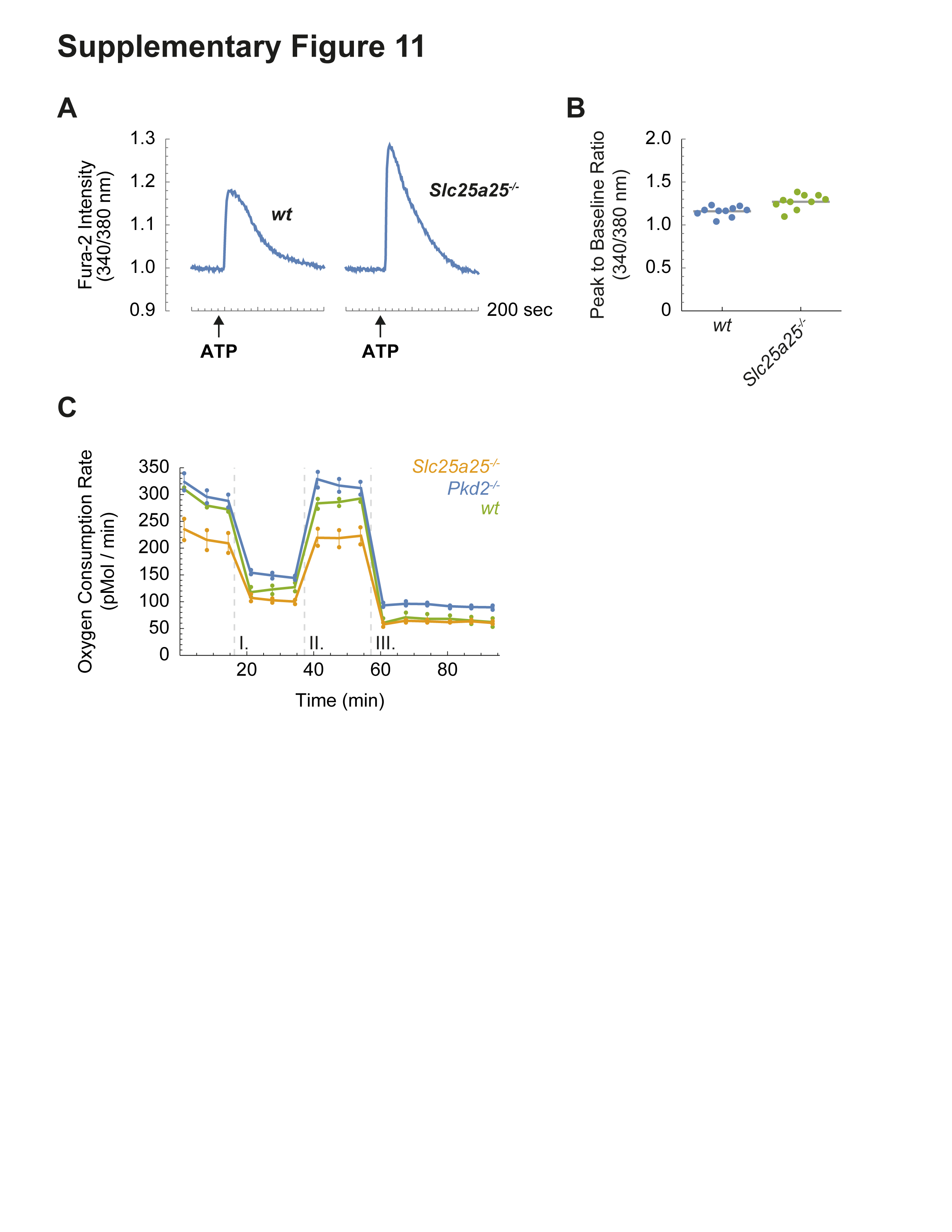

Supplement: S11 Fig — (A) Fura-2-AM-based intracellular Ca2+ measurement of ATP-induced (1 μM) Ca2+ transients in wt and Slc25a25−/− mIMCD3 cells. (B) No significant differences were observed (n = 10). (C) Oxygen consumption rates were measured at basal conditions and after injection of oligomycin (I.), FCCP (II.), and antimycin A plus rotenone (III.) at the indicated time points. The results are presented as mean ± standard error of the mean (n = 6). For numerical values see S1 Data. FCCP, carbonyl cyanide 4-(trifluoromethoxy)phenylhydrazone; Fura-2-AM, Fura-2-acetoxymethyl ester; wt, wild-type. (TIF) [file pbio.2005651.s011.tif]

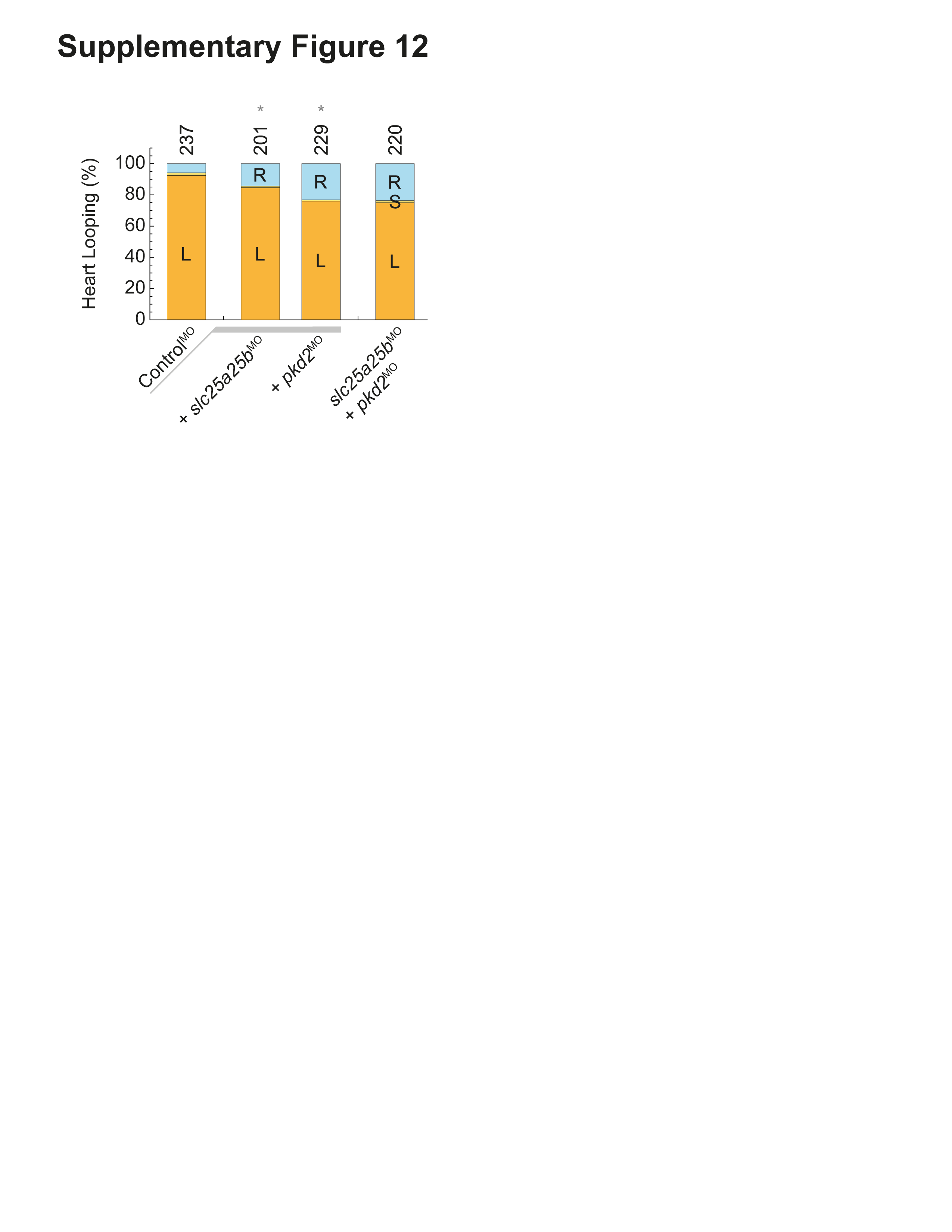

Supplement: S12 Fig — Individual application of slc25a25bMO and pkd2MO caused a significant increase in heart looping defects (*P = 0.01 and *P = 7 × 10^−7^, respectively). Parallel knockdown of slc25a25b and pkd2 did not aggravate the phenotype. Injection volume was approximately 2 nl comprising 5 ng ControlMO, 1.5 ng slc25a25bMO + 2.5 ng ControlMO, 2.5 ng pkd2MO + 1.5 ng ControlMO, or 2.5 ng pkd2MO + 1.5 ng slc25a25bMO. For numerical values, see S1 Data. (TIF) [file pbio.2005651.s012.tif]
