# Supplementary material for: The mitochondrial transporter SLC25A25 links ciliary TRPP2 signaling and cellular metabolism
Source: PLoS Biol. 2018 Aug 6;16(8):e2005651. doi: 10.1371/journal.pbio.2005651 (PMC6095617; doi:10.1371/journal.pbio.2005651)
Supplement: S1 Table — (DOCX) [file pbio.2005651.s013.docx]

**S1 Table. Changed metabolites observed for *Pkd2^-/-^* and *Slc25a25^-/-^* genotypes.**

| Metabolite | HMDB ID | Function / Pathway | References |
| --- | --- | --- | --- |
| Ratio increased | | | |
| taurine | HMDB00251 | Non-proteinogenic, essential, sulphur containing amino acid. Implicated in development and degenerative processes of neuronal system. | [1-3] |
| choline phosphate | HMDB01565 | Intermediate in biosynthesis of amino acids glycine, serine and threonine, and glycerophospholipids. Involved in fetal lung development. | [4,5] |
| 2-methyl-butyrylcarnitine (C5) | HMDB00378 | Short-chain acyl carnitine involved in amino acid metabolism of valine, isoleucine and leucine. Elevation of 2-methylbutyrylcarnitine suggests a deficiency of a dehydrogenase specific for isobutyryl-CoA. | [6] |
| Ratio decreased | | | |
| 3-(4-hydroxyphenyl)-lactate | HMDB00755 | Tyrosine metabolite which can act as inhibitor of ROS generation in mitochondria. | [7] |
| proline | HMDB00162 | Proteinogenic amino acid essential for collagen structure. |  |
| N-acetylalanine | HMDB00766 | Substrate for Guanine nucleotide-binding proteins. |  |
| alanine | HMDB00161 | One of the most important amino acids released by muscle, functioning as a major energy source. |  |
| N-acetylserine | HMDB02931 | Mostly found as an N-terminal amino acid directing acetylated proteins to ATP-ubiquitin-dependent proteasome. |  |
| N-acetylmethionine | HMDB11745 | Metabolite of amino acid methionine. |  |
| gamma-glutamylalanine | HMDB29142 | Dipeptide, an intermediate of protein metabolism. |  |

HMDB ID is a unique identifier of a given metabolite [8]. Pathway assignment is derived from HMDB.

**Reference**

1. Honda T. Amino acid metabolism in the brain with convulsive disorders. Part 3: Free amino acid patterns in cerebrospinal fluid in infants and children with convulsive disorders. Brain Dev. 1984;6(1):27-32. PubMed PMID: 6329017.

2. McNulty JA, McReynolds HD, Bowman DC. Pineal gland free amino acids and indoles during postnatal development of the rat: correlations in individual glands. J Pineal Res. 1990;9(1):65-73. PubMed PMID: 2231273.

3. Junyent F, Romero R, de Lemos L, Utrera J, Camins A, Pallas M, et al. Taurine treatment inhibits CaMKII activity and modulates the presence of calbindin D28k, calretinin, and parvalbumin in the brain. J Neurosci Res. 2010;88(1):136-42. doi: 10.1002/jnr.22192. PubMed PMID: 19658200.

4. Tokmakjian S, Haines DS, Possmayer F. Pulmonary phosphatidylcholine biosynthesis. Alterations in the pool sizes of choline and choline derivatives in rabbit fetal lung during development. Biochim Biophys Acta. 1981;663(2):557-68. PubMed PMID: 7213786.

5. Lee M, Han SS. Choline phosphate potentiates sphingosine-1-phosphate-induced Raf-1 kinase activation dependent of Ras--phosphatidylinositol-3-kinase pathway. Cell Signal. 2002;14(4):373-9. PubMed PMID: 11858945.

6. Vockley J, Ensenauer R. Isovaleric acidemia: new aspects of genetic and phenotypic heterogeneity. Am J Med Genet C Semin Med Genet. 2006;142C(2):95-103. doi: 10.1002/ajmg.c.30089. PubMed PMID: 16602101; PubMed Central PMCID: PMCPMC2652706.

7. Beloborodova N, Bairamov I, Olenin A, Shubina V, Teplova V, Fedotcheva N. Effect of phenolic acids of microbial origin on production of reactive oxygen species in mitochondria and neutrophils. J Biomed Sci. 2012;19:89. doi: 10.1186/1423-0127-19-89. PubMed PMID: 23061754; PubMed Central PMCID: PMCPMC3503878.

8. Wishart DS, Knox C, Guo AC, Eisner R, Young N, Gautam B, et al. HMDB: a knowledgebase for the human metabolome. Nucleic acids research. 2009;37(Database issue):D603-10. doi: 10.1093/nar/gkn810. PubMed PMID: 18953024; PubMed Central PMCID: PMCPMC2686599.
